# Enhancing Data Protection in Dynamic Consent Management Systems: Formalizing Privacy and Security Definitions with Differential Privacy, Decentralization, and Zero-Knowledge Proofs

**DOI:** 10.3390/s23177604

**Published:** 2023-09-01

**Authors:** Muhammad Irfan Khalid, Mansoor Ahmed, Jungsuk Kim

**Affiliations:** 1 Department of Information and Electrical Engineering and Applied Mathematics, University of Salerno Fisciano, 84084 Fisciano, Italy; 2 ADAPT Centre, Innovative Value Institute, Maynooth University, W23 A3HY Maynooth, Ireland; mansoor@comsats.edu.pk; 3 Research Laboratory, Cellico Inc., Seongnam-si 13449, Gyeonggi-do, Republic of Korea; 4 Department of Biomedical Engineering, Gachon University, Seongnam-si 13120, Gyeonggi-do, Republic of Korea

**Keywords:** data protection, dynamic consent management, security, privacy by design, general data protection regulation (GDPR), differential privacy, blockchain

## Abstract

Dynamic consent management allows a data subject to dynamically govern her consent to access her data. Clearly, security and privacy guarantees are vital for the adoption of dynamic consent management systems. In particular, specific data protection guarantees can be required to comply with rules and laws (e.g., the General Data Protection Regulation (GDPR)). Since the primary instantiation of the dynamic consent management systems in the existing literature is towards developing sustainable e-healthcare services, in this paper, we study data protection issues in dynamic consent management systems, identifying crucial security and privacy properties and discussing severe limitations of systems described in the state of the art. We have presented the precise definitions of security and privacy properties that are essential to confirm the robustness of the dynamic consent management systems against diverse adversaries. Finally, under those precise formal definitions of security and privacy, we have proposed the implications of state-of-the-art tools and technologies such as differential privacy, blockchain technologies, zero-knowledge proofs, and cryptographic procedures that can be used to build dynamic consent management systems that are secure and private by design.

## 1. Introduction

Users are often concerned about how their personal data is used. In the view of Asghar et al. in [1], individuals want to be sure that any data they furnish is being used legally and exclusively for the purposes they have consented to. In addition, users should be capable of withdrawing their consent at any time. Consent is captured as a lawful record, which can serve as proof in disputes or legal proceedings. Steinsbekk et al. in [2] point out that when users give consent about their data, they constrain how their data can be used and transferred. These limitations help to safeguard their privacy and stop their data from being misused or transmitted without their consent. According to Almeida et al. in [3], given the evident digitization of our society, traditional static paper-based procedures for recording consent are no longer suitable. For instance, digitization is becoming increasingly crucial in the medical sector, and the use of digital consent is an incredible advancement to expedite research tasks that utilize confidential health data. Similarly, in the view of Ekong et al. in [4], a noticeable portion of sensitive private data gathered and used by scientists, corporations, and administrations generates unprecedented hazards to individual privacy rights and the security of personal information.

Vigt et al. in [5] and Gstrein et al. in [6] explained the legal aspects of consent by providing a few insights from the General Data Protection Regulation in Europe; according to them, as stated in the article 7 and specified further in recital 32 of the GDPR, consent can be characterized as “consent of the data subject signifies any voluntarily provided, explicit, declared and precise gesture of the data subject’s desires by which he or she, by a remark or by a transparent affirmative activity, denotes understanding to the processing of confidential data connecting to him or her”.

Overall, the definition of consent delivered by the GDPR stresses the significance of acquiring informed and voluntary consent from individuals to process their personal information, e.g., receiving consent from patients when performing any research-related tasks on their data. By doing so, organizations can demonstrate their commitment to respecting individual’s privacy rights and complying with the GDPR’s provisions for data protection. The GDPR provisions for data protection aim to ensure that individuals have control over their personal data and are aware of the extent to which their data are being used [5,6].

Vigt et al. in [5] and Gstrein et al. in [6] demonstrated further the design of a dynamic consent management system; they stated that when designing a DCMS, five prerequisites must be satisfied: (i) consent should be willingly disseminated, meaning that individuals should have the option to give or withhold consent freely and without coercion; (ii) consent must be explicit, indicating that it should be given through a specific and affirmative action or statement; (iii) consent must be informed, meaning that individuals should have access to all necessary information regarding the processing of their data before giving consent; (iv) consent must be precise, indicating that it should be specific to the particular processing activities and purposes for which it is being given, and (v) conceded consent must be cancellable, meaning that individuals should have the right to withdraw their consent at any time. The GDPR framework provides guidance and recommendations on how organizations handle and process individuals’ confidential data [5]. There can be numerous applications where dynamic consent management systems can be applied. However, in the existing literature, the leading research is being done on implementing dynamic consent management systems for the healthcare sector in order to create sustainable e-healthcare services with the implications of state-of-the-art tools, systems, and technologies.

### 1.1. Using Subject’s Confidential Data

Following the GDPR provisions about privacy by design regulations, Wolford et al. in [5,7] elaborated that in recital 40 of the GDPR, there are five lawful grounds for utilizing confidential details of data subjects. These are: (i) when the processing is necessary for the performance of a contract to which the data subject is a party or to take steps at the request of the data subject before entering into a contract; (ii) when the processing of private data is required to rescue lives; (iii) when processing is mandated to complete a duty associated with the general welfare; (iv) when obtaining consent from data subjects to utilize their data; and (v) when the data processing is needed to comply with any legal duty.

Hence, “consent” can be considered one of the most straightforward bases for processing the subject’s data. Wolford et al. in [7] discussed the implications of GDPR on clinical trials and highlighted the importance of obtaining valid consent from research participants. Their work emphasizes the need for transparency, clarity, and specificity in obtaining consent from participants and suggests that a DCMS can efficiently facilitate the process. Their paper provides insights into the practical aspects of implementing GDPR in the context of clinical trials and offers recommendations to researchers and regulators to ensure compliance with GDPR requirements.

### 1.2. Dynamic Consent

Dynamic consent aims to address the limitations of paper-based and static consent methods (e.g., paper-based methods to write the data subject’s preferences about her data utilization) that have been used for a long time. Tokas et al. in [8], devised a new term, “dynamic consent”. According to [8], the term dynamic consent originated from large-scale data collection in biomedical research where participants were formerly hired for a long time. Dynamic consent is an informed consent approach in which the user who gives her data by approval can control the flow of her data from the user’s perspective. Likewise, Goncharov et al. in [9] provided extended details about the dynamic consent. According to [9], dynamic consent has imposed granular consent in dealing with personal information.

Ideally, dynamic consent is a method of digital communication without having paper-based procedures in which the data and the consent used to be kept on paper. Wee et al. in [10] pointed out that a dynamic consent mechanism connects the data subject, data controller, and requester in a way that authorizes individuals to provide organized consent agreements on specific personal information and manage who can collect, access, and use their data for which purposes. So, using a dynamic consent-based mechanism, a data subject could track who collected her data and for which purposes. In [9,10], authors emphasize that dynamic consent mechanisms connect the data subject, controller, and requester to provide more control and organization over shared personal information. Dynamic consent permits individuals to control who can collect, access, and use their data for specific objectives and periods, enriching transparency and confidence in data processing activities.

### 1.3. Actors in a Dynamic Consent Management System

In a dynamic consent management system, there clearly are the following actors:

1. Data Subject (DS);
2. Data Controller (DC);
3. Data Auditor (DA);
4. Data Requester (DR).

**Figure 1:**
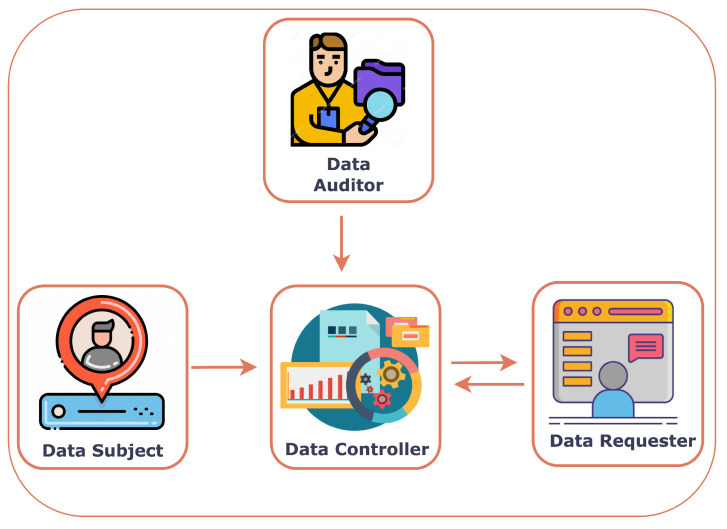
A typical dynamic consent management system.

Data Subject: A data subject is a person who submits her data and the corresponding consent to a data controller. She can be directly or indirectly identified.
Data Controller: A data controller is an entity working as a mediator and receiving information, possibly in some encoded form, from data subjects and providing data to data requesters. Notice that, in general, a data controller does not simply store the personal data of the data subject since this could already be a confidentiality violation. Indeed, notice that while a data subject might not want to share personal data with a DC, they might want to share personal data with a data requester through a data controller. A data controller is usually also accountable for keeping and correctly using the data subject’s consent.
Data Auditor: A data auditor is an entity with the authority and responsibility to inspect the operations performed by the data controller (e.g., to check if a DC is correctly implementing its role).
Data Requester: A data requester is an individual or organization that would like to process the private data of the data subject through a data controller. The data requester can be a researcher who wants to employ the subject’s data for research-associated tasks. Figure 1 depicts all the actors and their interactions in a dynamic consent management system.

### 1.4. Privacy by Design

Traditionally, abuses in data protection are punished and discouraged by the fact that laws explicitly address sanctions for the violators. More recently, there has been an extensive effort toward achieving appealing properties by design. This is a more demanding requirement aiming at guaranteeing that abuses are impossible. Belli et al. [11] discuss the significance of including privacy by design principles in developing and managing those systems that utilize data. Their work in [11] argues that privacy should not be treated as an afterthought to a component but incorporated into the design stage.

The notion of privacy by design strives to furnish technical and managerial solutions to safeguard the confidentiality of data subjects. Likewise, their work in [11] emphasizes the challenges developers and data controllers encounter in executing privacy by design, including balancing privacy problems with the demand for data collection and processing for honest purposes. The authors in [11] urge that privacy by design can be accomplished via a combined effort between designers, developers, and data protection professionals by entrenching privacy protections into the design of data systems.

### 1.5. Dynamic Consent Management Systems (DCMSs)

The existing literature has made significant efforts to define and explain the implications and benefits of dynamic consent management systems for enabling data subjects to manage their consent dynamically [12,13]. Considering this fact, Budin et al. in [14] demonstrated DCMS as a personalized online contact tool that encourages continuing two-way communication between the data requester and controllers and the data controller and subjects. To clarify further, Kaye et al. in [15] suggest that the DCMS can enable data subjects to modify their consent in response to changing circumstances, such as changes in the purpose of data processing or changes in the data subject’s preferences. In other words, a DCMS allows individuals to update their consent as their circumstances change, which can help ensure that their personal data is being processed in a way that is consistent with their wishes.

Spencer et al. in [16] suggested that a DCMS can enhance transparency and public trust by employing well-designed user interfaces and data infrastructures. Multiple methods exist for managing consent in real-world scenarios [17,18]. Hils et al. in [17] discuss a methodology for measuring the effectiveness of dynamic consent in research settings. Their paper provides an overview of dynamic consent, including using electronic consent forms and implementing DCMS. Likewise, Santos et al. in [18] focused on implementing dynamic consent in the context of health research. Their paper provides an overview of the critical considerations for implementing dynamic consent, including designing user-friendly interfaces, ensuring transparency and accountability, and addressing ethical and legal issues. Considering currently used DCMSs, we mention MyData-Operators in [19] which provides a DCMS in healthcare and research settings, Onetrust in [20], and Ethyca in [21], which both offer a privacy management platform that includes DCMS. Onetrust and Ethyca discuss DCMS focusing on privacy, but only Ethyca, in [21], claims to provide privacy by design.

Additionally, in the white papers of [19,20,21], it is evident that the data controller stores data subject’s personal information and consent in a centralized database. Furthermore, in those white papers, there are no precise definitions of privacy and security properties, e.g., how the confidentiality of involved partie’s data (e.g., data subject’s data) will be preserved when sharing that subject’s data with the data controller and requester. Likewise, there is no precise information on how the unforgeability of the outputs for each honest entity is protected in the presence of diverse adversaries. Apart from confidentiality and unforgeability, there are no explicit details regarding how the availability and auditability of their given DCMS will be fulfilled in the face of challenges from various foes. Therefore, the claims of [21] about privacy by design remain excessively vague. Hence, as discussed in Asghar et al.’s work in [22], creating a DCMS that guarantees the security and privacy of the actors in the presence of adversaries is a big challenge.

### 1.6. Access Controls in Dynamic Consent Management Systems

Access control is an essential security mechanism for organizations to manage sensitive data. It involves restricting access to resources based on the identity and privileges of users, as well as enforcing policies that determine what actions are allowed or denied [23]. In the context of DCMSs, access control is crucial to maintaining the confidentiality and integrity of DS, DC, DA, and DR. Access control mechanisms, policies, and systems can be used to ensure that only authorized individuals can access or modify sensitive data and that any such access or modification is recorded and audited. Dynamic access control policies are more flexible than traditional access control mechanisms and adapt to changing requirements and access patterns. One major challenge in DCMSs is the need to balance the privacy of DSs with the legitimate needs of DC, DA, and DR to access the subject’s data. Access control mechanisms in DCMS could be designed to ensure that only authorized players can access the DS’s data while also providing sufficient transparency and accountability to ensure the DS’s data is used appropriately without violating the access control preferences of DSs.

In this ongoing Ph.D. research project, “Security and Privacy in DCMS”, whose central part is this work, we are also interested in using access control mechanisms for DCMS to explore the possibility of developing new techniques and policies that balance the competing interests of privacy and access to the desired data. For example, for a DR in a DCMS, the possibility of using role-based access controls can be employed to explore the use of multi-factor authentication or biometric-based access control mechanisms to increase the security of DCMS. Meanwhile, there could be a focus on developing access control policies that consider the dynamic nature of DCMS, including changes in the types of data being collected, differences in access patterns, and changes in the entities who require access to the DSs data. Additional investigations into implementing access controls in DCMS can enhance these system’s security, privacy, flexibility, and adaptability to cater to the evolving needs of both users and organizations.

### 1.7. Why Previous Works in Access Controls Are Insufficient to Resolve the Problems Faced by This Paper

The study carried out in this work is about data protection in DCMSs, when there are diverse internal attackers who can attack the system in various ways. Previous work in access control is insufficient for managing the complex challenges posed by data protection in DCMSs. Fine-grained access control policies that can be modified dynamically based on the context of the data requests are needed, along with tools for organizing and managing consent for data usage and establishing trust between involved parties, i.e., the data subject, controller, auditor, and the requester. Traditional access control mechanisms depend on static policies and do not deliver ways to manage and enforce consent or set trust, making them ineffective for handling the exceptional challenges of DCMSs. New access control mechanisms explicitly developed for DCMSs are required to guarantee the integrity and confidentiality of the data subject while enabling data sharing to meet the needs of all parties involved. Due to these obvious facts, the previous work in access control is insufficient to resolve the problem faced by this paper.

### 1.8. Research Questions This Work has Addressed

- What data protection issues are related to all the dynamic consent management system entities?
- What is the current state of the art in dynamic consent management systems regarding implementations considering privacy and security properties?
- Which procedures are run by two parties, i.e., DS, DC-DC, DR, where two parties interact to perform a joint computation in a dynamic consent management system, and what do both parties get in output?
- How do we state the correctness of a dynamic consent management system when there are no attacks on such systems, and what does each party get from these systems upon giving input?
- What vital security and privacy properties are crucial to ensure robust data protection for all the dynamic consent management system entities, and how to formally define these security and privacy properties?
- How to address the essential tools and techniques to ascertain the robustness and resilience against diverse attacks in dynamic consent management systems, and how the latest tools and techniques could be utilized in dynamic consent management systems?

### 1.9. Our Contributions

This paper proposes a model in terms of formally defining a sequence of resilient properties to assess the security and privacy of DCMSs, taking into account the data collection procedures, data storage, data audit, and data processing by the data subject, data controller, data auditor, and data requester, respectively. To investigate the need for security and privacy for such a DCMS, we have elaborated on how the integrity and confidentiality of all the actors of a DCMS should be preserved in the presence of adversaries who will attack the system.

Summing up, this work includes the following contributions:

- We have proposed a model in terms of defining what a dynamic consent management system (DCMS) is, and then we have described all the procedures that are run by each of the entities in a dynamic consent management system while interacting with each other. In this procedure’s description model, we have mainly stressed the procedures where two actors interact with each other to perform a joint computation.
- We elaborate on what ideally (i.e., without considering adversaries) a DCMS should provide to the involved parties. Here, we mainly address the correctness of a dynamic consent management system when there are no attacks, and the system provides correct and accurate output to each interacting party.
- We have discussed the definitions aiming at capturing privacy and security properties for a dynamic consent management system in the presence of adversaries that can attack the system. We have formally discussed privacy and security properties to have precise insights into the robustness of a dynamic consent management system (DCMS).
- Next, we emphasize limits in both definitions and constructions of existing dynamic consent management systems (DCMSs) from the state of the art. We highlighted with appropriate clarifications how the existing studies in dynamic consent management systems lack security and privacy for all the entities of these dynamic consent management systems.
- We propose the use of specific techniques and tools that could be used to mitigate security and privacy risks in dynamic consent management systems (DCMSs).
- We have mainly studied the potential of differential privacy to increase the robustness of the dynamic consent management systems in terms of fulfilling the needs of all the involved entities such as Data Subject and Data Requester. The code for these experiments is available here *Differential-Privacy-for-DCMS*: https://github.com/Khalid-Muhammad-Irfan/Differntial-Privacy-for-DCMS (accessed on 3 July 2023). Furthermore, we have also discussed the implications of zero-knowledge proofs along with blockchains where only an abstract entity Data Controller should be decentralized to have enhanced data protection for all the entities.

The rest of this paper is organized as follows. The detailed critical analysis of the existing works in DCMS constructions has been specified in Section 2. The motivations for considering security and privacy properties for DCMS have also been illustrated in Section 2. Section 3 elaborates on the model we have defined in terms of specifying precise procedures run by each party in DCMS to interact with each other. Section 4 describes the attacker’s model. Section 5 is about the proposed formal definitions of security and privacy properties in DCMSs. The mitigations under the proposed sketch of formal privacy and security definitions have been elaborated in Section 6. The brief discussions about this article are described in Section 7. The conclusions and the future recommendations in light of this work have been described in Section 8. The list of acronyms is explained in Table 1.

## 2. State-of-the-Art on Existing Solutions for Dynamic Consent Management Systems

Though various solutions exist for implementing a dynamic consent management system, we briefly review the most related ones in Section 2. Specifically, we will focus on stressing the main weaknesses of existing dynamic consent management system solutions concerning the security, privacy, and functional requirements addressed in Section 3, Section 3.1 and Section 5. There can be numerous applications where DCMS can be applied. However, in the existing literature, the leading research is being done on implementing DCMS in the healthcare sector. In Table 2, we point to the prior works in available dynamic consent management systems. We have found that none or only a few satisfy particular security and privacy property definitions concerning the proposed dynamic consent management systems, i.e., the definition of confidentiality related to the involved player’s data/consent (e.g., data subject) when sharing with the data controller and requester the definition of unforgeability of the output for honest parties.

The availability definition concerns the given dynamic consent management system (e.g., how proposed dynamic consent management systems ensure high availability with minimal downtime or disruption). The definition of auditability involves e.g., maintaining audit trails of consent-related activities, such as when consent was obtained, updated, or revoked and by whom. Apart from this, from Table 2, it is clear that we do not consider “security and privacy” as the complete, correct, and absolute definitions for all the underlying properties for data protection in the dynamic consent management system. Instead, when a DCMS is designed and developed, all the underlying definitions of security and privacy properties, e.g., “confidentiality, unforgeability, availability, auditability”, must also be explicitly discussed at each player’s level. Futuristic DCMSs must ensure these definitions of security and privacy from the design phase.

### 2.1. Explanation of the Referenced Paper(s) in Table 2

Merlec et al. in [12] proposed a smart contract-based DCMS, backed by blockchain technology, targeting personal data usage under GDPR. Merlec et al. developed a few design requirements in this work to explain the need for secure and confidential DCMS. In their work in [12], there are no definitions of the underlying security and privacy properties, e.g., no precise definition of the confidentiality of involved parties data (e.g., data subject) when sharing it with the data controller and requester. This work did not consider the unforgeability of the output for the honest parties. There are no considerations about the possible adversaries and attacks (e.g., DoS and DDoS attacks) while stating the performance evaluation of the given DCMS. Likewise, there are no definitions of the availability (e.g., how high availability of the proposed DCMS is ensured with minimal downtime to the data subject, controller, and processor). Although Merlec et al. employed blockchain in their contribution, there needs to be formal or informal information on how the auditability of the entire DCMS is ensured and which entity is responsible for backtracking things. As a result, this study in [12] did not consider the security and privacy properties we defined in Section 5 concerning the proposed DCMS.

Prictor et al. in [24] propose a logic model framework for evaluating and reporting DCMS effectiveness. Though with the provided framework, Prictor et al. explained various benefits of DCMSs, e.g., regarding DCMS as a precise means of consent management compared to other means of consent like paper-based and static consent procedures. Nevertheless, Prictor et al. did not consider the privacy and security aspects of the proposed DCMS framework, e.g., there is no information about the confidentiality of the involved actor’s data in the DCMS. Also, there are no considerations about the possible adversaries, internal and external, to the system. The demonstration of the unforgeability of the outputs for the involved honest players, i.e., the data subject, the data controller, and the data user, needed to be included in this work. Authors in [24] failed to define availability and auditability concerning the given DCMS precisely. Thus, it is apparent that the study did not consider the security and privacy properties we have described in Section 5 concerning the proposed DCMS.

In this work, Casass et al. in [25] propose an approach, “Encore”, which is initialized after a project to ensure the patient’s participation in clinical and medical research. In their work, authors have demonstrated use cases where a data subject sends data and consent to the controller, and then a requester requests that subject’s data and consent from the controller. For all three use cases, authors in [25] claimed the definitions of our proposed security and privacy properties concerning each use case, such as confidentiality, unforgeability, and availability. Still, no precise information could demonstrate how the confidentiality of involved parties’ data (i.e., data subject) is achieved when sharing with the data controller and requester. Similarly, there was no precise definition of the unforgeability of the output for the honest actors considering numerous adversaries in the system. In this work, Casass et al. failed to explain the availability of their given system, i.e., how the proposed DCMS will manage to get high availability when numerous data subjects access the system to update their shared consent and data. Hence, the confidentiality, unforgeability, and availability descriptions were excessively vague. Moreover, in this work [25], auditability concerning the given DCMS is claimed to be achieved at each use case level (the data subject, controller, requester). Still, Casass et al. could not provide sufficient evidence in their work that this claim is valid. Therefore, it is clear that the paper [25] failed to address the security and privacy properties that we described in Section 5 regarding the proposed DCMS.

In this work, Genestier et al. in [26] discuss the possibilities of using hyper ledger fabric blockchain for DCM and to address privacy and security challenges in the eHealth domain where a patient is granting and revoking access to her personnel data. Their work assesses blockchain as a tool to tackle the existing problems in consent management, e.g., centralized storage and sharing of data and consent of a data subject by a trusted third party. Despite blockchain implications in this work, Genestier et al. did not give any security and privacy evaluations that can ascertain the definitions of our proposed model. For instance, in the work by Genestier et al., we could not find any suitable description of the confidentiality of the actor’s data and the unforgeability related to the output of each honest actor in the proposed system. Similarly, availability and auditability definitions of the proposed DCMS were not considered in the work by Genestier et al. In light of our detailed description of the security and privacy properties necessary for a robust DCMS in Section 5, it is evident that work of Genestier et al. in [26] did not take these properties into account for the proposed DCMS. Therefore, the work lacks a thorough evaluation of the system’s security and privacy features.

Rupasinghe et al. in [27] described a privacy-preserving consent model architecture using blockchain to facilitate patient data acquisition for clinical data analysis. Their proposed architecture is a high-level illustration of blockchain-based DCMS. In their work, the details about the confidentiality of the involved parties’ data, e.g., the confidentiality of patient data/consent and unforgeability concerning the output for each honest entity, were found missing. For example, in this work [27], there was no formal or informal evidence through which one can comprehend that involved parties in the proposed blockchain-based DCMS are enjoying confidentiality and unforgeability. Meanwhile, the definitions of availability (e.g., how high availability is concerning proposed DCMS and how efficiency is ensured when the number of entities accessing DCMS is increasing exponentially) pertaining to the proposed DCMS were not considered in [27]. Utilizing blockchain technology, Rupasinghe et al. claimed that auditability is achieved where parties, e.g., patients, can audit logs to ensure auditability. Still, we could not find any relevant evidence about auditability in this work. Given the specific security and privacy properties that we have outlined for a robust DCMS in Section 5, it is evident that Rupasinghe et al. did not incorporate these properties into the proposed DCMS. As a result, the study falls short in evaluating the system’s security and privacy measures.

Jaiman et al. in [28] proposed an Ethereum blockchain-based DCMS to control access to individual health data, where smart contracts represent separate consent and authorize the requester to request and access health data from the patients. The proposed DCMS consists of patients as data subjects, a data-sharing agreement-based smart contract, and a data processor (who wants to process the subject’s data). In their work, the confidentiality of patient data was not discussed precisely when shared with the processor and stored onto the blockchain in plain text form (e.g., plain-text data shared onto a blockchain that poses various privacy constraints). The unforgeability of the output of each involved honest player (when the number of foes can forge the outcome) is not considered at all. Furthermore, Jaiman et al. failed to define the proposed DCMS’s availability (e.g., how the availability of patient data is ensured for the processor while keeping it on a public blockchain and how Ethereum blockchain-based DCMS is scalable when many processors request it through invoking the data-sharing smart contract?). Likewise, in this work [28], auditability descriptions concerning the opted DCMS were found to be quite vague. There is no precise information on how patients can perform audit trials to know how the processor uses their data. The paper [28] in question failed to address the security and privacy properties that we specified for an effective DCMS in Section 5 in the context of the proposed DCMS. Therefore, the study does not provide a comprehensive evaluation of the security and privacy features of the system.

This paper by Albanese et al. in [29] presents SCoDES, an approach for trusted and decentralized control of DCM in clinical trials based on blockchain technology. This work utilized hyper ledger fabric blockchain to remove the trust in third parties while managing the consent dynamically in clinical trials. Their work focuses on creating a web-based platform using a private blockchain network where entities, e.g., patients, can dynamically control their consent and data. In their work, Albanese et al. did not provide clear definitions of confidentiality regarding how involved parties’ data (e.g., patient’s data) are ensured while considering numerous adversaries and also when these sensitive data are shared with the data processor. Furthermore, the unforgeability of the output of the honest parties, i.e., data subject and processor, was not explored and disseminated clearly. Meanwhile, the authors of [29] could not describe the precise definitions of availability concerning the proposed DCMS. In this work [29], auditability claimed to be acquired through blockchain, but we could not find any evidence that this was true. The paper [29] under review failed to incorporate the security and privacy properties we have carefully defined for an effective DCMS in Section 5.

This work by Mamo et al. in [30] provides a construction of the DCMS that should be conceived in the bio-banking sector. Intrinsically, their work presents a web portal backed by a hyper ledger composer blockchain framework and a hub connecting various Malta bio-banking stakeholders. In their work, the precise definitions of confidentiality of involved parties’ data (e.g., data of customers and banks) and the unforgeability of the output of honest customers and other involved stakeholders were not demonstrated. Likewise, the definition of availability (e.g., how the system is enjoying availability while adding more nodes onto the fabric network) of the proposed DCMS was missing under what we have conceived and presented in our model, as we stated in Section 5. Furthermore, the auditability was claimed to be achieved using the hyper ledger composer framework. Still, we did not find suitable proof to elaborate on how an actor such as a customer (whose data are with the banks) can backtrack auditability in the proposed DCMS. The analyzed paper did not consider the security and privacy properties specified for the proposed DCMS in Section 5. Consequently, the work by [30] did not adequately address the requirements for ensuring data confidentiality, unforgeability, availability, and auditability, which are crucial for a robust DCMS.

In this work, Bhaskaran et al. in [31] described designing and implementing a smart contract-based DCMS driven by double-blind data sharing on the hyper ledger fabric platform. Bhaskaran et al. have demonstrated how a (know your customers) KYC application builds around their proposed DCM model to address the needs of the banks while meeting regulatory requirements. In their work, the unforgeability of the outputs for the involved honest actors (e.g., a bank and a customer) is claimed to be achieved. Still, we did not find any precise information about the claim’s validity. Apart from that, the description of the confidentiality of entities’ data (e.g., data of a customer while sharing with a bank) was not considered. Bhaskaran et al. failed to define the availability of their blockchain-based network (when the number of customers increases exponentially in permissioned setup) under what we have conceived and proposed in our model as we stated in Section 5. Using blockchain, the work in [31] claims that auditability concerning actors (e.g., customer can audit trials to know how her data are used and by whom) is being achieved. Still, we need help finding sufficient evidence that this claim is valid in this paper. The reviewed paper did not consider the security and privacy properties that we defined in Section 5 for the proposed DCMS.

Rupasinghe, in her doctoral thesis in [13], presented a blockchain-based DCMS for the secondary use of electronic medical records. Her suggested method for DCMS has almost all the components that should satisfy the possible constraints set by data governance authorities such as GDPR and HIPAA. Rupasinghe tried to create a few design goals for this DCMS to highlight the importance of design activity while developing a blockchain-based DCMS. In the doctoral thesis by Rupasinghe, the proposed DCMS only consists of a prototype implemented using hyper ledger fabric blockchain. However, the precise definitions of underlying important security and privacy properties are missing, such as how the confidentiality of involved parties’ data (e.g., data subject) is achieved when sharing with the data steward and requester. How is the integrity of the outputs for each honest party, such as data subject, steward, and requester, guaranteed considering adversaries? Instead, Rupasinghe assumed that confidentiality and unforgeability are acquired by employing blockchain in her given DCMS. Apart from that, Rupasinghe did not provide definitions for availability concerning her proposed DCMS (e.g., how high availability of the proposed DCMS is ensured with minimal downtime to the data subject, steward, and requester? In addition, how scalable and efficient is the proposed system when the number of data subjects increases exponentially?). As Rupasinghe utilized blockchain technology, there were claims about achieving auditability, but we could not witness any proof of this claim. Our defined security and privacy properties for the proposed DCMS, as elaborated in Section 5, were not considered in this Ph.D. thesis research context.

Kim et al. in [32] have proposed a DCMS backed by blockchain technology based on a rule-set management algorithm for managing healthcare data called Dynamichain. Their proposed approach was implemented where the exercise management healthcare company provided health management services based on data from the data provider’s hospital. For the implementation, the work in [32] has utilized hyper ledger fabric blockchain where known entities interact. In this work by Kim et al., there is no information about the performance analysis that can state the security and privacy of the proposed DynamiChain model. For example, in work given by Kim et al., there are no definitions of the underlying security and privacy properties such as how the confidentiality of involved parties data is being achieved (e.g., the confidentiality of data provider data in DynamiChain) when shared with the hospital, and data utilizer. Likewise, no information about the unforgeability of the output for honest parties in the system (like the data provider, the data controller, and the data utilizer) is guaranteed when foes can change the output for these honest entities. Meanwhile, in [32], the authors did not provide definitions for availability concerning DynamiChain (e.g., how high availability of the proposed DCMS is ensured with minimal downtime to actors such as data providers, or how scalable and efficient the proposed system is when the number of parties who are accessing the proposed DynamiChain increased exponentially?). As Kim et al. have utilized hyper ledger blockchain technology to attain decentralization, there were claims about achieving auditability about actors (e.g., data providers can backtrack things and see who used their data and for what purpose). Still, we could not witness any actual proof of this claim. Therefore, Kim et al. failed to consider the security and privacy properties we defined for the proposed DCMS in Section 5.

### 2.2. Motivation behind Considering Security and Privacy Properties for DCMSs

The reason for concentrating on these security and privacy properties of DCMSs is that various researchers have determined them as critical obstacles to the widespread adoption of DCMSs [33]. Moreover, legislation worldwide has emphasized the significance of designing future systems that handle public data with privacy and security in mind, such as the GDPR by the European Union [5]. These properties are also established on previously published best techniques. For example, the European Health Data Space (EHDS) [34] has highlighted the need for secure and private system designs that can be used for data sharing. Table 3 describes previous works that aimed to enforce privacy and security by design in any system that utilizes public data.

## 3. Defining Dynamic Consent Management System (DCMS)

A dynamic consent management system is a complex system that involves multiple procedures to perform various tasks related to managing a subject’s consent and data. Here in our model, we are not interested in knowing what an actor does on its own locally in DCMS, but we are interested in learning the interactions of two parties (e.g., interactions between DS and DC, interactions among DC and DA, and interactions between DR and DC). So, in our model, we are mainly interested in procedures where two parties interact to perform a joint computation. When two parties give input to the DCMS, a procedure executes among those two parties as the result of those inputs. After the execution of that particular procedure, both parties get a possible output from the DCMS.

Below, we describe the procedures run by the involved actors of a DCMS.

1. **DS Sharing Data/Consent:**
  In this procedure, DS and DC are involved since DS wants to share its data/consent, and DC is supposed to update its state according to the interaction with DS.
  - **Adding Data/Consent**
    Here, DS would like to share some data and consent with DC.
    ***Input for DS:*** Data, consent, and access control policies related to data and consent.
    ***Input for DC:*** The current state associated to DS.
    ***Output for DS:*** A receipt about the executed procedure (both in case the outcome has been positive or negative).
    ***Output for DC:*** An updated state associated to DS.
  - **Updating Data/Consent**
    In this scenario, DS and DC are involved since DS shared some data and consent with DC and now DS wishes to make some updates to her shared data and consent.
    ***Input for DS:*** A receipt issued by DC upon receiving DS data/consent.
    ***Input for DC:*** The current state associated to DS.
    ***Output for DS:*** A receipt about the updated executed procedure (both in case the outcome has been positive or negative).
    ***Output for DC:*** The updated state associated to DS.
2. **DA Verifying DC:**
  Here, DA and DC are involved since DA wants to audit whether DC has done all the operations related to DS’s data/consent honestly.
  ***Input for DA:*** Credentials proving the right to audit the DC.
  ***Input for DC:*** The current states associated to all DS.
  ***Output for DA:*** A receipt about the verified information (both in case the outcome has been positive or negative).
  ***Output for DC:*** The current state associated to DS.
3. **DR Requesting Data/Consent from DC:**
  Here, DR would like to access DS’s data and consent. Notice that due to our discussion in Section 3.1, regarding the choice of discouraging the direct communication between DR and DS, DR must ask DC for DS’s data/consent.
  - **Requesting Data/Consent:**
    In this procedure, DR and DC are involved. DR wishes to get DS’s data/consent to perform his research-related tasks. DC is supposed to update its state according to the interactions with DR.
    ***Input for DR:*** Credentials proving the right to access DS’s data/consent.
    ***Input for the DC:*** The current state associated to DS.
    ***Output for DR:*** A receipt about the executed procedure (both in case the outcome has been positive or negative).
    ***Output for DC:*** The updated state associated to DS.

### 3.1. Correctness of a Dynamic Consent Management System (DCMS)

A DCMS is said to satisfy a correctness requirement if whenever the procedures are run correctly, the outputs obtained by every actor in every phase are always correct according to the inputs provided. We remark that for correctness, it is assumed that there are no attacks on a DCMS. We also remark that in our model, we excluded a direct communication between DS and DR. Indeed, in a DCMS that relies on direct communication between DS and DR, there can be several potential issues. Typically, a DS would like to passively share its data, avoiding time-consuming interactions with all possible (i.e., legitimate and non-legitimate ones) DRs who are interested in obtaining its data. DS typically would simply like to give consent to a DC that is specialized in dealing with DRs. Such consent can even be generic in the sense of specifying categories of those DRs who can access data.

In the correctness of a DCMS, every player is giving the correct input to run the desired procedures described in Section 3. As a result, those players get the correct output from the system. The precise requirements for the correctness of a DCMS are defined below. In all previous procedures that have been elaborated in Section 3, whenever DS, DC, DA, and DR behave honestly, they get the following outputs:

- **DS Adding data/consent:**
  ***Output for DS:*** Receipt about the correctly executed procedure (in result of correct input).
  ***Output for DC:*** The updated state associated to DS.
- **DS Updating data/consent:**
  ***Output for DS:*** Receipt about the correctly executed procedure (in result of correct input).
  ***Output for DC:*** The Updated state associated to DS.
- **DA Verifying DC:**
  ***Output for DA:*** A receipt about the verified information (in result of correct input).
  ***Output for DC:*** The current state associated to DS.
- **DR Requesting Data/Consent from DC:**
  ***Output for DR:*** A receipt about the executed procedure (in result of correct input).
  ***Output for DC:*** The updated state associated to DS.

## 4. Attackers Model

Keeping in mind the properties we defined in Section 5 for the resilience of a DCMS, all the actors can attack the DCMS in numerous ways.

- **Possible Attackers:** It has been evident from the literature that the threats to every system start for two types of reasons. An internal user with legal system access, such as a data subject, controller, auditor, or requester, will access the subject’s data. On the other hand, there is an external agent. Although external agents are not allowed to enter the systems, there is always a higher likelihood that a critical opponent from the external environment can offer various threats to the system. In DCMSs, we have recognized four kinds of actors who can play with the system.
- **Data Subject:** In a DCMS, the data subject providing her data and giving consent could also be permitted to access and interact with the system in diverse ways. This could be for recreational purposes or other motivations.
- **Data Controller:** In a DCMS, the data controller is one of the most critical actors with a substantial stake in the system’s operation. The data controller has access to the system throughout the process, from data collection and storage of consent in the server to monitoring all system activities from a central position. However, the data controller could also pose a risk as a potential attacker who may attempt to compromise the system. For instance, data controllers may unlawfully access and sell data subject’s confidential information or share it with unauthorized organizations. Apart from that, since the data controller is a centralized entity that is processing data/consent, it is crucial to avoid a single point of failure due to a corrupted data controller; thus, this must be an abstract entity which should be decentralized only. We will be giving a follow-up work to this paper that will essentially consist of studying the techniques to decentralize the data controller in a DCMS.
- **Data Auditor:** A data auditor can be a potential DCMS attacker. Although the primary role of a data auditor is to ensure that the data controller complies with the policies and regulations related to data privacy and security, he may still have access to sensitive data that could be used for malicious purposes. Suppose a data auditor gains unauthorized access to the system. In that case, they may be able to manipulate data, compromise the integrity of the audit trail, or interfere with the consent management process.
- **Data Requester:** The data requester may have access to data and consent of the data subject through DCMS, and there is a severe risk that he may misuse the subject’s data for harmful purposes. For example, a requester could use the subject’s data for identity theft, fraud, or other illegal activities. In addition, the data requester may not have the necessary security measures to protect the data, leading to the potential exposure of the subject’s data to unauthorized parties. This can significantly harm the data subject, including through financial loss, reputational damage, and emotional distress.

## 5. Defining Security and Privacy in Dynamic Consent Management Systems

In a DCMS, both privacy and security properties concern the protection of honest players in the presence of some misbehaving players. The goal is to preserve the confidentiality of the honest player’s data and the unforgeability of outputs for each honest party.

We will more concretely discuss the security and privacy of a DCMS, considering more specific properties such as Confidentiality, Unforgeability, Availability, and Auditability.

**Figure 2:**
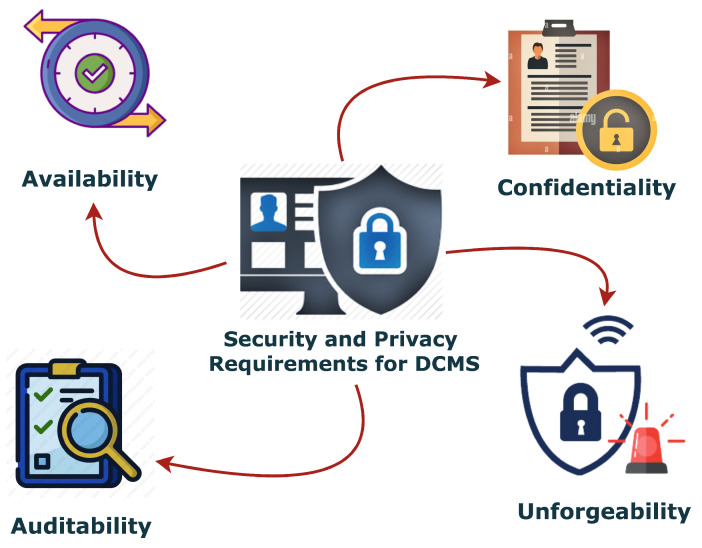
Proposed resilient properties to make a robust dynamic consent management system.

As described in Figure 2, a robust DCMS should enjoy the following properties:

1. 1. **Confidentiality:** The confidentiality property in a dynamic consent management system should be guaranteed, e.g., for an honest DS when DC is dishonest, when DA is dishonest, or when DR is dishonest, or maybe when all are dishonest. The system provides the minimum amount of data required by the requester according to the consent released by the data subject, and unauthorized access to the data of any actors in a DCMS should be infeasible. Confidentiality of honest parties (i.e., DS, DC, DA, DR) is defined as follows:
  - **Confidentiality for DS:**
    According to the procedures defined in Section 3, in a DCMS parties are involved in procedures and a DS provides her data in input to some of those procedures (e.g., sharing or updating data/consent). As motivated when discussing the threat model in Section 4, we can expect players to misbehave and thus deviate from the expected steps specified by the procedures (e.g., providing incorrect inputs) with the goal of obtaining unauthorized information. In the context of a DCMS ensuring the confidentiality of the honest DS from a dishonest DC, we can describe the confidentiality for the DS with the help of an experiment DCPrivKdcms,DC,Π(n) that will illustrate the formal specification of the definition. In addition, in the following experiment, we assume that in a dynamic consent management system only the adversary DC operates within polynomial time. We acknowledge that DC is an adversary and may select the encrypted message from DS with a probability slightly more significant than 1/2.
    **Experiment:**
    We write Pr[DCPrivKdcms,DC,Π(n), to denote the experiment being run with security parameter *n*:
    Pr[DCPrivKdcms,DC,Π(n)=1]≤1/2+negl(n), The experiment is defined for any private-key encryption scheme Π=〈gen,share,access〉, any adversary DC, and any value n for the security parameter:
    The adversarial indistinguishability experiment DCPrivKdcms,DC,Π(n)
    − In the DCMS, the adversary DC, is given input ($1^{n}$), and outputs a pair of messages $m_{0}$, $m_{1}$ with |$m_{0}$| = |$m_{1}$|.
    − A key *k* is generated by running Gen ($1^{n}$), and a uniform bit b∈(0,1) is chosen. Cipher-text *c*←$Enc_{k}(m_{b}$) is computed in a dynamic consent management system and sent to the adversary DC. We refer to *c* as the challenge cipher-text, which is a message essentially consisting of the data/consent of the data subject (DS).
    − DC outputs a bit $b^{\prime }$.
    − The output of the experiment is defined to be 1 if $b^{\prime }$ = *b*, and 0 otherwise. If DCPrivKdcms,DC,Π(n)=1, we say that DC succeeds.
    Here, we are considering adversary DC running in polynomial time, and we accept that the DC might determine the encrypted message from the data subject (DS) with a probability negligibly better than 1/2. In the context of a dynamic consent management system ensuring the confidentiality of the honest data subject from a dishonest data controller, we can describe it as follows:
    **Formal Definition:** A DCMS Π=〈gen,share,access〉 has indistinguishable encryptions in the presence of a dishonest DC, or is DC−IND secure, if, for all probabilistic polynomial-time dishonest data controllers (DCs), there is a negligible function negl such that, for all *n*:
    Pr[DCPrivKdcms,DC,Π(n)=1]≤1/2+negl(n), Here, the idea is that an adversary DC must not be able to get information about the plain text that essentially contains the data/consent of the data subject (DS) encrypted using an encryption key *K* and is in cipher-text form. IND secure refers to the Indistinguishable encryptions, which is a property of the security definition, and Π represents the dynamic consent management system (DCMS) comprising three algorithms:
    **Gen:** The key generation algorithm generates a secret key for the data subject (DS) and the data controller (DC) to securely communicate and establish trust.
    **Share:** The data sharing algorithm allows the DS to share her data/consent with the DC securely. This algorithm takes the data/consent and secret key *K* as input and produces a ciphertext consisting of encrypted data.
    **Access:** The access algorithm enables DC to access and process the subject’s (DSs) shared data/consent. The access algorithm takes the cipher-text, which is actually encrypted data/consent, and the secret key *K* as input and produces the plain-text data of DS.
    **Indistinguishable encryptions:** Indistinguishable encryptions are the property of the security definition. A DCMS Π is said to have indistinguishable encryptions if it is computationally hard for a dishonest DC to differentiate between two instances of encrypted data by observing the encrypted data.
    **Probabilistic polynomial-time dishonest data controllers (DCs):** The dishonest DCs are computationally bounded algorithms that attempt to break the security of a DCMS by giving incorrect input to one of the procedures run by the DS to share/update her data/consent. DCs can access public algorithms (Share and Access) and perform polynomial-time computations.
    **Negligible function (negl):** A negligible function is a minimal function that approaches zero as its input size increases. In the context of DCMS security analysis, a negligible function represents a level of DCMS security that is considered practically impossible to break.
    Pr[DCPrivKdcms,DC,Π(n)=1]≤1/2+negl(n): This inequality expresses the DC−IND security property of DCMS. It states that for all probabilistic polynomial-time dishonest DC, the probability that the adversary correctly determines whether a given encrypted data corresponds to a specific data/consent is at most 1/2 plus a negligible function negl(n). In other words, the advantage of the dishonest data controllers (DCs) distinguishing the shared data is minimal, approaching zero as *n* (the security parameter) increases.
    The above description defines the requirement for a dynamic consent management system to have indistinguishable encryptions, ensuring the confidentiality of the data subjects (DSs) data/consent from dishonest data controllers DCs. Fundamentally, in the above scenario, DS is an honest entity giving correct inputs to procedures as specified in Section 3, and expecting correct output from the dynamic consent management system. In this situation, the confidentiality property of DCMS will come into play to preserve an honest DS’s sensitive data/consent when DC, DA, or DR may act as adversaries while deviating from the procedures. Since DA and DR also have direct or indirect contact with DS’s data/consent, they might also be believed as possible foes for the confidentiality of DS’s data/consent. Hence, to satisfy confidentiality for DS, a DCMS should have strong security and privacy tools in place to ensure that DS’s data/consent are not misused by dishonest parties. Confidentiality can be foreseen in a given construction of DCMS by employing useful tools and techniques such as advanced encryption schemes (as the indistinguishability property can be achieved using advanced encryption schemes), zero-knowledge proofs, and differential privacy.
  - **Confidentiality for DC:**
    According to the above procedures in Section 3, the data controller (DC) takes part in procedures the Data Auditor (DA) runs to verify the DC. Initially, DA runs a procedure to verify the DC. The DC behaves as an honest party by giving the procedure the correct input (i.e., the current state of all data subjects (DSs)). Keeping in view the experiment that is explained in defining the confidentiality of an honest data subject (DS), we are now stating the confidentiality for an honest DC when a DA may misbehave as follows:
    **Experiment:**
    We write Pr[DAPrivKdcms,DA,Π(n), to denote the experiment being run with security parameter n:
    Pr[DAPrivKdcms,DA,Π(n)=1]≤1/2+negl(n), The experiment is defined for any private-key encryption scheme Π=〈gen,share,access〉, any adversary DA, and any value *n* for the security parameter:
    The adversarial indistinguishability experiment DAPrivKdcms,DA,Π(n)
    − The adversary DA, is given input ($1^{n}$), and outputs a pair of messages $m_{0}$, $m_{1}$ with |$m_{0}$| = |$m_{1}$|.
    − A key *K* is generated by running Gen ($1^{n}$), and a uniform bit b∈0,1 is chosen. Ciphertext *c*←$Enc_{k}(m_{b}$) is computed and given to the adversary DA. We refer to *c* as the challenge ciphertext.
    − DA outputs a bit $b^{\prime }$.
    − The output of the experiment is defined to be 1 if *b* = *b*, and 0 otherwise. If DCPrivKdcms,DC,Π(n)=1, we say that DA succeeds.
    Here, we are considering adversary DA running in polynomial time, and we accept that the DA might determine the encrypted message, which is the data/consent of all DSs from the DC with a probability negligibly better than 1/2. In the context of a dynamic consent management system ensuring the confidentiality of the honest DC from a dishonest DA, we can describe it as follows:
    **Formal Definition:** A DCMS Π=〈gen,share,access〉 has indistinguishable encryptions in the presence of a dishonest DA, or is DA−IND secure, if for all probabilistic polynomial-time dishonest data auditors DAs, there is a negligible function negl such that, for all *n*:
    Pr[DAPrivKdcms,DA,Π(n)=1]≤1/2+negl(n), Here, the idea is that an adversary DA must not be able to get information about the plain-text that essentially contains the data/consent of all DSs that is encrypted using an encryption key *K* and is in cipher-text form and being held by the honest DC. IND secure refers to the Indistinguishable encryptions, which is a property of the security definition, and Π represents the DCMS comprising three algorithms: Π=〈gen,share,access〉.
  - **Confidentiality for DA:**
    As specified in Section 3, the DA is supposed to verify whether all the operations performed by the DC related to the DS’s data/consent have been performed honestly or not. For these verifications, for instance, when the DA wishes to verify the DC, he runs a procedure in DCMS where the DA gives correct input that is essentially his right to access and audit the DC. Likewise, the DC needs to feed input to this procedure by stating the current state associated with all DSs. In this procedure, the DA acts as an honest player and provides the correct credentials to audit the DC. Still, at the same time, DC can deviate from the procedure by feeding the wrong input.
    So, the DC is one of the potential attackers for the confidentiality of DA’s data. The confidentiality property of a dynamic consent management system will ensure the inputs and outputs of an honest DA when DC is acting as an adversary. Being a dishonest party, DC will be sharing the wrong data/consent records with the DA. Keeping in view the experiment that is explained in defining the confidentiality of an honest DS, we are now stating the confidentiality for an honest DA as follows:
    **Experiment:**
    We write Pr[DCPrivKdcms,DC,Π(n), to denote the experiment being run with security parameter n:
    Pr[DCPrivKdcms,DC,Π(n)=1]≤1/2+negl(n), The experiment is defined for any private-key encryption scheme Π=〈gen,share,access〉, any adversary DC, and any value *n* for the security parameter:
    The adversarial indistinguishability experiment DCPrivKdcms,DC,Π(n)
    − The adversary DC, is given input ($1^{n}$), and outputs a pair of messages $m_{0}$, $m_{1}$ with |$m_{0}$| = |$m_{1}$|.
    − A key *K* is generated by running Gen ($1^{n}$), and a uniform bit b∈0,1 is chosen. Ciphertext c ←$Enc_{k}(m_{b}$) is computed and given to the adversary DC. We refer to *c* as the challenge ciphertext.
    − DC outputs a bit $b^{\prime }$.
    − The output of the experiment is defined to be 1 if $b^{\prime }$ = *b*, and 0 otherwise. If DCPrivKdcms,DC,Π(n)=1, we say that DC succeeds.
    Here, we are considering adversary DC running in polynomial time, and we accept that the DC might determine the encrypted message from the DA with a probability negligibly better than 1/2. In the context of a DCMS ensuring the confidentiality of the honest DA from a dishonest DC, we can describe it as follows:
    **Formal Definition:** A DCMS Π=〈gen,share,access〉 has indistinguishable encryptions in the presence of a dishonest DC, or is DC−IND secure, if, for all probabilistic polynomial-time dishonest DCs, there is a negligible function negl such that, for all *n*:
    Pr[DCPrivKdcms,DC,Π(n)=1]≤1/2+negl(n), Here, the idea is that an adversary DC must not be able to get information about the plain text that essentially contains the confidential information of an honest DA. IND secure refers to the Indistinguishable encryptions, which is a property of the security definition, and Π represents the DCMS comprising three algorithms: Π=〈gen,share,access〉.
  - **Confidentiality for DR:**
    According to the procedures described in Section 3, DR runs a procedure to ask for the DS’s data/consent from DC. Here, we assume that the DR is an honest entity feeding correct input, i.e., correct credentials proving the right to access the DS’s data/consent. Meanwhile, the DR expects the DC to also give correct input (i.e., the current state associated with DS) to the procedure. If, somehow, the DC is not providing correct input, the DC is deviating from the procedure run by the DR. Hence, the DC is a possible adversary who can misuse the information of the DR. Since the DR would be sharing his credentials to access the DS’s data/consent, the DC may use this information.
    In addition, the DR does not want to share his access request information with any other player, i.e., DS and DA, but lawful DC should see the requests from DR. Now, if the DC misbehaves, then the DR’s data would also be compromised by the dishonest DC. The confidentiality property of DCMS will ensure that the requests made by honest DRs have been protected efficiently in the sense that both the input and output of a genuine DR shall be maintained by the confidentiality property of DCMS. Keeping in view the experiment that is explained in defining the confidentiality of an honest DS, we are now stating the confidentiality for an honest DR as follows:
    **Experiment:**
    We write Pr[DCPrivKdcms,DC,Π(n), to denote the experiment being run with security parameter *n*:
    Pr[DCPrivKdcms,DC,Π(n)=1]≤1/2+negl(n), The experiment is defined for any private-key encryption scheme Π=〈gen,share,access〉, any adversary DC, and any value *n* for the security parameter:
    The adversarial indistinguishability experiment DCPrivKdcms,DC,Π(n)
    − The adversary DC, is given input ($1^{n}$), and outputs a pair of messages $m_{0}$, $m_{1}$ with |$m_{0}$| = |$m_{1}$|.
    − A key *K* is generated by running Gen ($1^{n}$), and a uniform bit b∈0,1 is chosen. Ciphertext *c*←$Enc_{k}(m_{b}$) is computed and given to the adversary DC. We refer to *c* as the challenge ciphertext.
    − DC outputs a bit $b^{\prime }$.
    − The output of the experiment is defined to be 1 if $b^{\prime }$ = *b*, and 0 otherwise. If DCPrivKdcms,DC,Π(n)=1, we say that DC succeeds.
    Here, we are considering adversary DC running in polynomial time, and we accept that the DC might determine the encrypted message from the DR with a probability negligibly better than 1/2. In the context of a DCMS ensuring the confidentiality of the honest DR from a dishonest DC, we can describe it as follows:
    **Formal Definition:** A DCMS Π=〈gen,share,access〉 has indistinguishable encryptions in the presence of a dishonest DC, or is DC−IND secure, if for all probabilistic polynomial-time dishonest DCs, there is a negligible function negl such that, for all *n*:
    Pr[DCPrivKdcms,DC,Π(n)=1]≤1/2+negl(n), Here, the idea is that an adversary DC must not be able to get information about the plain text that essentially contains the confidential information of an honest DR. IND secure refers to the Indistinguishable encryptions, which is a property of the security definition and Π represents the DCMS comprising three algorithms: Π=〈gen,share,access〉.

2. **Unforgeability:**
  The dynamic consent management system should be able to provide correct outputs to honest players, i.e., DS, DC, DA, and DR, even in the presence of adversaries who can misbehave during the executions of the procedures specified in Section 3. We now consider the unforgeability for each player of a DCMS.
  **Experiment:** *Dynamic-Consent-OutputForge A*, Π(n)
  - Gen ($1^{n}$) is run to obtain keys (pk,sk) for the DCMS.
  - Adversary *A* is given pk and access to the DCMS, including the player DS.
  - The adversary *A* interacts with the DCMS, providing inputs and receiving outputs.
  - The adversary *A* outputs a forged output for the honest entity, i.e., DS, DC, DA, or DR. The adversary *A* succeeds if and only if the forged output is accepted as a valid output by the DCMS.
  - In this case, the output of the experiment is defined to be 1.
  **Definition:** Dynamic-Consent-Output-Unforgeability for an honest party:
  A DCMS Π=〈gen,sign,vrfy〉 is considered output secure for the honest entity like DS, DC, DA, and DR, if for all probabilistic polynomial-time adversaries *A*, there exists a negligible function negl such that the probability of the adversary *A* successfully forging the output of the honest entity in the experiment Dynamic-Consent-OutputForge *A*,Π(n) is negligible:
  Pr[Dynamic−Consent−Output−Forge*A*, Π(n) = 1] ≤negl(n)
  Here *A* is denoted as dishonest party that might act as an adversary. So, a DCMS is an output secure for an honest entity (DS, DC, DA, and DR) if the probability of an adversary *A* forging a valid output that a legitimate entity would receive is extremely low (negligible) when faced with a computational adversary *A* who has access to the DCMS functionalities. The output refers to the correctness of an honest entity in the form of receipts or any other relevant information generated by the DCMS. We now map the above experiment to each of the involved parties’ interactions in a DCMS below:
  - **Unforgeability for DS:**
    Here, DS is an honest party providing correct input to the procedures executed with DC. Being an honest party, DS has the privilege of getting the correct output from the system, even if any adversary, such as a DC, tries to forge the output of DS. In the defined procedures in Section 3, while interacting with DC, the outcomes for DS are receipts about the executed procedures that state that data/consent have been received, stored, and processed correctly by the DC. Even though other players in DCMS (such as DA, DR, and DC) will still be accessing DS data/consent directly or indirectly, the output (essentially the correctness of DS’s data/consent) must be guaranteed by the DCMS. In this experiment, two parties, i.e., DS and DC, are involved since both these parties are interacting with each other in a couple of procedures that have been stated in Section 3.
    **Experiment:** *Dynamic-Consent-OutputForge DC*, Π(n)
    − Gen ($1^{n}$) is run to obtain keys (pk,sk) for the DCMS.
    − Adversary DC is given pk and access to the DCMS, including the player DS.
    − DC interacts with the DCMS, providing inputs and receiving outputs.
    − The adversary DC outputs a forged output for the honest DS. DC succeeds if and only if the forged output is accepted as a valid output by the DCMS.
    − In this case, the experiment’s output is defined to be 1.
    **Formal Definition:** Dynamic-Consent-Output-Unforgeability for honest DS:
    A dynamic consent management system Π=〈gen,sign,vrfy〉 is considered output secure for the honest DS if, for all probabilistic polynomial-time adversaries like DC, there exists a negligible function negl such that the probability of the adversary DC successfully forging the output of DS in the experiment Dynamic-Consent-OutputForge DC,Π(n) is negligible:
    Pr[Dynamic−Consent−Output−ForgeDC, Π(n) = 1] ≤negl(n)
    A DCMS is an output secure for the honest DS if the probability of a dishonest DC forging a valid output that DS would receive is extremely low (negligible) when faced with a computational adversary DC with access to the system functionalities and DS. The output refers to the correctness of DS’s data and consent in the form of receipts or any other relevant information generated by the DCMS.
  - **Unforgeability for DC:**
    In the defined procedures in Section 3, the possible outputs for DCs are the current and updated states associated with all DSs in DCMS. Here, we have to protect the correctness of the output of DC when DS, DA, or DR misbehaves. Unforgeability for DC means that output for an honest DC should be correct in the sense that when a DC is honest, then the output for DC should be correct regardless of the honesty or dishonesty of other players, i.e., DS, DA, and DR that are interacting with DC. Since DC is considered an honest player, he must be guaranteed the correct output from DCMS even if the other players misbehave.
    So, here we are concerned with protecting the unforgeability of outputs for a DC being an honest party. Hence, the states corresponding to all DSs must not be altered or modified by any misbehaving player in DCMS. Since DC is first interacting with DS to receive data/consent, being an honest party providing the correct input to the procedures, DC must be ensured to get the correct output from the system. In the sense that even if DS tries to modify the output for DC, the DCMS should provide the correct output to DC.
    In this experiment, two parties, i.e., DC and DA, are involved since both these parties are interacting with each other in a couple of procedures that have been stated in Section 3.
    **Experiment:** *Dynamic-Consent-OutputForge DA*, Π(n)
    − Gen ($1^{n}$) is run to obtain keys (pk,sk) for the DCMS.
    − Adversary DA is given pk and access to the DCMS.
    − Adversary DA interacts with the DCMS, providing inputs and receiving outputs.
    − The adversary then outputs a forged output for the honest DC. Hence, the DA succeeds if and only if the forged output is accepted as a valid output by the DCMS.
    − In this case, the experiment’s output is defined to be 1.
    **Formal Definition:** Dynamic-Consent-Output-Unforgeability for honest DC:
    A dynamic consent management system Π=〈gen,sign,vrfy〉 is considered output secure for the honest DC, if for all probabilistic polynomial-time adversaries like DA, there exists a negligible function negl such that the probability of the adversary DA successfully forging the output of DC in the experiment Dynamic-Consent-OutputForge *A*,Π(n) is negligible:
    Pr[Dynamic−Consent−OutputForgeDA, Π(n) = 1] ≤negl(n)
    In simpler terms, a DCMS is an output secure for the honest DC if the probability of an adversary DA forging a valid output that DC would receive is extremely low (negligible) when faced with a computational adversary DA who has access to the system functionalities and DC. The output refers to the correctness of DCs data in the form of receipts or any other relevant information generated by the DCMS.
  - **Unforgeability for DA:**
    Here, we need to protect the correctness of the output of the DA when DC misbehaves. As detailed in Section 3, the DA is an honest entity feeding correct input while executing a procedure with DC. So, DA must be ensured to get correct output from DCMS even if DC misbehaves, providing incorrect input to the procedure. The output for DA is the receipt that states all the procedures related to DS’s data/consent have been performed honestly by the DC. For a DA, unforgeability mainly refers to any audit report generated by the DCMS that should be genuine and accurately reflect the system’s state and cannot be tampered with or modified by any malicious party such as DC or DR.
    Since adversaries such as DC or DR may try to forge the output for the DA, i.e., making modifications in audit records, the integrity property of DCMS will ensure that an honest DA should always get a correct output, even if other interacting players try to forge the output for DA by giving incorrect input to the procedure with DA. In this experiment, two parties, i.e., DA and DC, are involved since both these parties are interacting with each other in a couple of procedures that have been stated in Section 3.
    **Experiment:** *Dynamic-Consent-OutputForge DC*, Π(n)
    − Gen ($1^{n}$) is run to obtain keys (pk,sk) for the DCMS.
    − Adversary DC is given pk and access to the DCMS.
    − Adversary DC interacts with the DCMS, providing inputs and receiving outputs.
    − The adversary then outputs a forged output for the honest DA. DC succeeds if and only if the forged output is accepted as a valid output by the DCMS.
    − In this case, the experiment’s output is defined to be 1.
    **Formal Definition:** Dynamic-Consent-Output-Unforgeability for honest DA:
    A dynamic consent management system Π=〈gen,sign,vrfy〉 is considered output secure for the honest DA, if for all probabilistic polynomial-time adversaries like DC, there exists a negligible function negl such that the probability of the adversary DC successfully forging the output of DA in the experiment Dynamic-Consent-OutputForge A, Π(n) is negligible:
    Pr[Dynamic−Consent−Output−ForgeDC, Π(n) = 1] ≤negl(n)
    In simpler terms, a DCMS is output secure for the honest DA if the probability of an adversary DC forging a valid output that DC would receive is extremely low (negligible) when faced with a computational adversary DC with access to the system functionalities and DA. The output refers to the correctness of DAs data in the form of receipts or any other relevant information generated by the DCMS.
  - **Unforgeability for DR:**
    Here, the DR is an honest party running a procedure with the DC to have the DS’s data/consent. Being a legitimate party, the DR is providing the correct input to the procedure. Still, there is a possibility that the DC may give an incorrect input to the procedure, i.e., providing the wrong states associated with DS. In this case, the integrity of outputs for an honest DR will be protected by the DCMS. Hence, the correctness of the output of DR shall be protected when the DC misbehaves.
    The possible outputs for the DR are required DS’s data/consent according to his credentials. We need to preserve the integrity of the outputs for a DR to be an honest party. In the sense that even though adversaries (such as DA and DC) are present inside the DCMS, a DR must be able to get the correct data/consent for which he requested to a DC. If, for instance, an adversary tries to modify the output for the DR, the DCMS should implement robust tools and techniques that can hinder these malicious modifications. More formally, the unforgeability for a DR states that any data/consent as an output obtained from the DCMS must be genuine and accurate and cannot be tampered with or modified by any malicious or unauthorized player.
    In this experiment, two parties, i.e., DR and DC, are involved since both these parties are interacting with each other in a couple of procedures that have been stated in Section 3.
    **Experiment:** *Dynamic-Consent-OutputForge DC*, Π(n)
    − Gen ($1^{n}$) is run to obtain keys (pk,sk) for the DCMS.
    − Adversary DC is given pk and access to the DCMS.
    − Adversary DC interacts with the DCMS, providing inputs and receiving outputs.
    − The adversary then outputs a forged output for the honest DR. DA succeeds if and only if the forged output is accepted as a valid output by the DCMS.
    − In this case, the experiment’s output is defined to be 1.
    **Formal Definition:** Dynamic-Consent-Output-Unforgeability for honest DR:
    A dynamic consent management system Π=〈gen,sign,vrfy〉 is considered output secure for the honest DR, if for all probabilistic polynomial-time adversaries like DC, there exists a negligible function negl such that the probability of the adversary DC successfully forging the output of DR in the experiment Dynamic-Consent-OutputForge *A*,Π(n) is negligible:
    Pr[Dynamic−Consent−Output−ForgeDC, Π(n) = 1] ≤negl(n)
    Hence, a DCMS is output secure for the honest DR if the probability of an adversary DC forging a valid output that DR would receive is extremely low (negligible) when faced with a computational adversary DC with access to the system functionalities and DR. The output refers to the correctness of DRs data in the form of receipts or any other relevant information generated by the DCMS.

3. **Availability:**
  Access to the dynamic consent management system should only be granted to authenticated actors (DS, DC, DA, and DR). The DCMS must be designed so that it should be highly available. Highly available means that there should be no interruption or downtime in the system, even due to hardware or software failures, which may result in delays or errors in data processing and communication among peers. If there are any delays or interruptions, these can ultimately impact the privacy and security of the data and the players involved in DCMS. High availability of the DCMS must be ensured to remain accessible to actors at all times.
  The system should be designed to be resilient to denial of service (DoS) and distributed denial of service (DDoS) attacks, ensuring that it remains up and running even during a large-scale attack. There should be no server downtime to ensure that actors such as DSs have timely and reliable access to their data/consent. DSs should be able to view, update, or delete their data/consent and provide or withdraw consent for collecting, using, and disclosing their data. DSs should also be informed about data processing, including privacy or security breaches. Adversaries such as DS, DC, DA, and DR should not be able to compromise the system’s high availability.
4. **Auditability:**
  Each DC in a dynamic consent management system is associated with a DA that has access to information specific to that DC. Auditability is an essential aspect of transparency that ensures the system’s smooth operation. The DCMS must be auditable to ensure that all processes within the system are functioning correctly. Auditability allows for the verification of actions taken by all actors within the system. Essentially, auditability is a technique to construct reliable DCMS. To facilitate this, the DCMS should maintain detailed event logs for all operations performed by each actor. These logs will enable actors such as DS and DC to trace audit logs to verify if DRs have used their data following consent granted by the DS.

## 6. Mitigations to Avoid Potential Adversaries and Attacks on DCMSs

Although the specific mitigation plan may vary depending on the type of attack, we have developed a risk reduction strategy based on the proposed threats, which we explained in Section 4 for a dynamic consent management system. The following Section outlines the mitigation strategies for the DCMS.

**Tools and techniques that can be used to mitigate those threats that affect previous work:**

In Section 4 and Section 5, we discussed various security and privacy risks associated with DCMS. To mitigate these threats, we propose specific tools and techniques that can be employed. As mentioned in Section 5, we have identified Confidentiality, Unforgeability, Availability, and Auditability as crucial properties that can be utilized to address these risks. In Section 5, we have elaborated on these properties in detail. In this Section, we comprehensively describe the relevant tools and techniques that can be used to mitigate the threats to DCMS under each of these defined properties.

**Figure 3:**
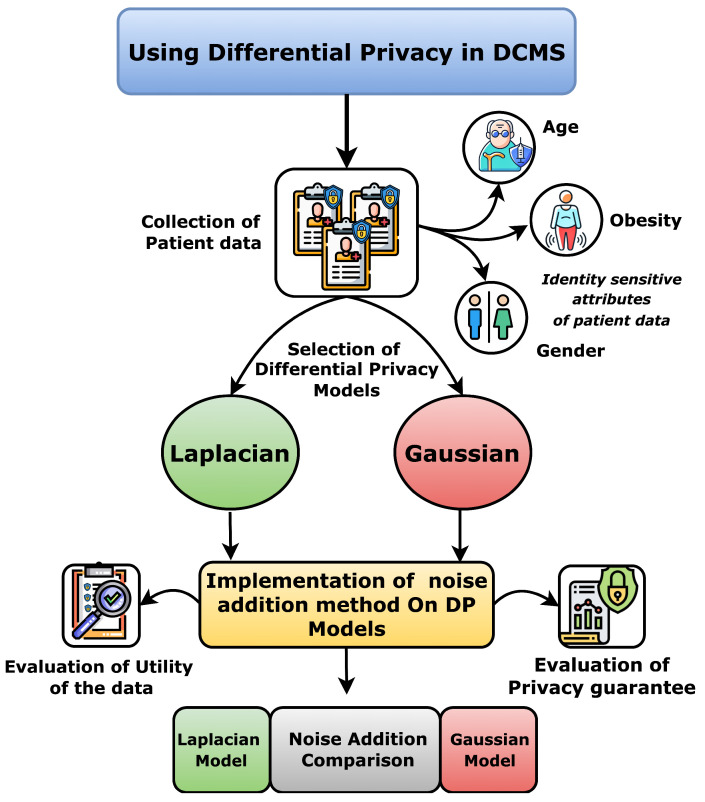
Differential privacy implications in dynamic consent management system settings.

**Figure 4:**
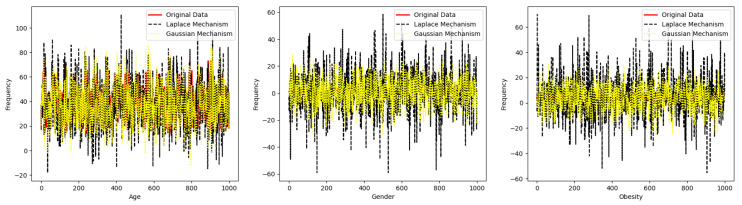
Data privacy and utility trade off among Gaussian and Laplacian noise addition mechanisms at ϵ = 0.1.

**Figure 5:**
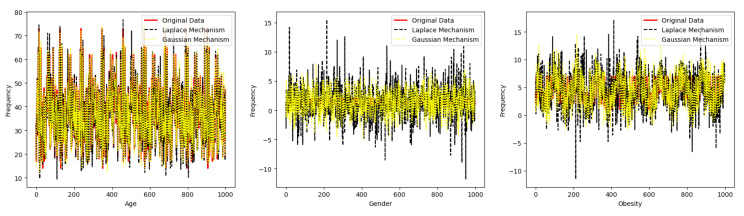
Data privacy and utility trade off among Gaussian and Laplacian noise addition mechanisms at ϵ = 0.5.

**Figure 6:**
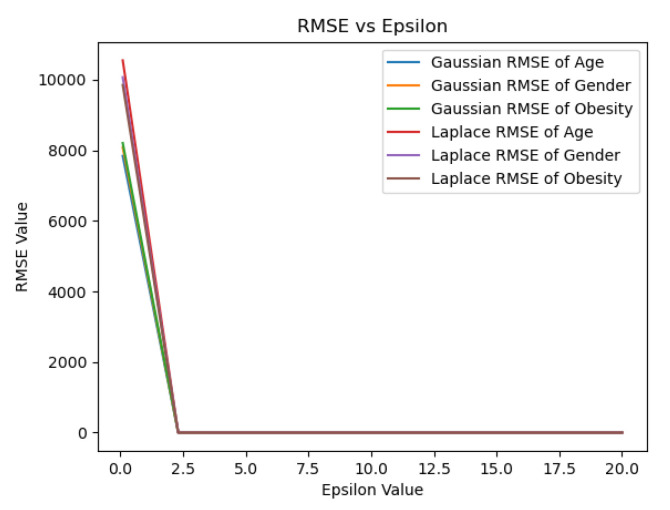
RMSE values for Laplacian and Gaussian mechanisms concerning patient data processing in dynamic consent management system.

1. **Confidentiality:**
  The confidentiality of the honest parties (i.e., DS, DC, DA, DR) must be protected effectively in DCMS when some dishonest parties can compromise the confidentiality of the honest parties. Tools and techniques (mentioned below) could be vital in ascertaining the confidentiality of the involved parties data (e.g., the subject’s data) when shared with the data controller and requester. Tools and techniques are: *Anonymization, Pseudonymization, and Data Masking, Differential Privacy, Zero Knowledge Proofs, Homomorphic Encryption, Secure Multi-party Computation.* Below, we only discuss that tool and the technique that seems promising in mitigating severe threats in DCMS.
  - ***Differential Privacy (DP):***
    According to [45], differential privacy assures data subjects (DSs) (whose data will be utilized for research-related tasks by DRs) that when a DS provide consent to authorize her healthcare data to be employed by any researcher, data subjects (DSs) will not have severe outcomes by permitting their data to be dispersed to the data requesters (DRs). The data controller (DC) must also obey what data subjects (DSs) have agreed to about their data usage. Differential privacy ϵ can be used to make DCMSs more resilient to severe attacks. For instance, DSs can utilize data perturbation mechanisms, i.e., Laplacian or Gaussian, to make their data noisy before sharing with DCs. This noise addition will strictly safeguard the privacy of the DS’s data, and at the same time, DRs could also be able to analyze this noisy data to perform their research tasks.
    However, when noise is added, there is a substantial trade-off between data privacy and its utility. Yet, DP can have features that, if carefully followed, mean this trade-off can be relaxed [46]. Moreover, there is often a parameter δ in differential privacy: the probability of information being leaked accidentally. More formally, DP can be stated as: (δ, ϵ Differential Privacy). L here is the randomized method that meets (δ,ϵ) differential privacy even though there is (ϵ) > 0, (δ) > 0, in such a way that:
    (1) $$Pr[\left(D_{1}\epsilon S\right]\leq exp\left(\epsilon \right)Pr[\left(D_{2}\leq S\right]\;+\delta ,$$
    where ϵ and δ are privacy parameters: the closer to 0 they are, the higher the privacy level is. The (δ, ϵ)-differential privacy is often achieved by adding noise to the output of a function f(DS). The above equation stands for each range (L) and for any data sets $\left(D_{1}\right)$ and $\left(D_{2}\right)$ which vary just one portion and everything *S*⊆ Range (L). In a randomized system, *L* achieves (ϵ,0)-differential privacy while δ=0. After a thorough investigation into integrating differential privacy framework in DCMS to achieve robust privacy for DS data while confirming its required utility for the DR, we have found a significant trade-off between the utility and privacy of DS data. This trade-off concerns the protection of DSs’ privacy when sharing sensitive data containing personal information with the DR while ensuring that the data stays deferentially private to allow DRs to examine it for research purposes. Taking these aspects into consideration, we have made the following findings.
**Using Differential Privacy in Dynamic Consent Management System:**
    Differential privacy has been employed broadly to protect the privacy of data owners/patients in data sharing scenarios [47,48,49,50], even without DCMS. However, no research currently has used DP in a DCMS setting to ensure robust privacy for data subjects (DSs) while preserving the utility of data for data requesters (DRs). DCMS provides individuals with more control over their data, allowing them to dynamically set preferences on how their data is shared and with whom [14,16]. Combining DP with DCMS can give DSs more control over their data privacy. Data subjects can set different privacy levels (i.e., varying values for privacy budget ϵ) for different data types, specifying the purpose of data sharing and revoking consent at any time. This setting allows DSs to make more informed decisions about how their data are being used and to have greater transparency and control over the data-sharing process. Implementing DP in a DCMS can also increase trust in data-sharing by providing mathematical guarantees of DSs’ privacy protection. In summary, while DP has been used without a DCMS, using DP in a DCMS setting offers greater control and transparency for DSs over their data privacy and can increase trust in the data-sharing process. The noise level added to the data in DCMS can vary case by case.
  - **Contribution employing DP to concrete settings in DCMS:**
    Based on the available works on differential privacy frameworks such as local and global DP, we suggest that a DCMS can be used to map patient data management by hospitals (DCs) and the subsequent access of this data by researchers (DRs). In this setting, DSs can enjoy privacy while hospitals (DCs) can process and share sensitive data with DRs who need patient data for their research. By using DP in DCMS, DSs privacy can be maintained while allowing for the efficient processing and sharing of sensitive data. The precise and step-by-step depiction of the Differential Privacy implications can be seen in Figure 3.
  - **DS shares her data/consent with the DC, and DR requests for the DS Data to DC:**
    A patient (DS) shares their data after adding noise using either the Gaussian or Laplacian noise addition mechanism. The hospital (DC) receives and stores this data, aggregating multiple patients’ data in the DCMS database server. When a data requester (DR) requests the DS’s data, the DC checks its authenticity before sharing the accumulated and necessary data. Due to the intrinsic features of DCMS and the goal of ensuring security and privacy guarantees for DS’s data, no involved entities are trusted (as we explained in Section 4). So, we believe that DS must be able to share her data after adding sufficient noise so that no one can extract more than the required information from her data. We will now discuss the steps necessary to utilize DP in a DCMS setting, which will be considered one of the contributions of this work. Notice that, keeping the setting of DCMS in mind, here we are only taking patient data (DSs) that would be processed through DCMS. We also state that we have tested the possibility of using DP for (DSs) patient data so that the privacy of the patient’s data is ensured during its transmission to the hospital (DC) and finally to the researcher (DR) who would also be able to analyze this noisy data for his research-related tasks.
    Differential privacy is a framework for providing privacy in data analysis by adding noise to the data [45]. So, our primary goal is to protect the privacy of individual patient (DS) data while allowing a researcher (DR) to analyze the aggregated data properly. The available research studies in patient data privacy have considered differential privacy as one of the essential frameworks in healthcare since patient data are highly sensitive and must be protected to comply with regulations such as the General Data Protection Regulations (GDPR) in Europe [49]. Apart from that, it is important to note that testing differential privacy-based noise addition methods on patient data requires careful consideration of privacy regulations and ethical concerns as well [51]. To test various differential privacy-based noise addition methods on patient data, we followed the below steps:
    (a) ***Collection of patient data:***
      In the first step, we took a patient data set from Kaggle. This data set consisted of 25 attributes, i.e., patient ID, gender, obesity, etc. Intrinsically, healthcare data contains categorical data (such as diagnosis codes, laboratory tests ordered, and geographical information about the patients) and numerical data (such as age in years and duration of stay in hospital). So, there is a dire need for research to agree on a DP-based data pollution method to ascertain privacy and utility concerning patient data.
    (b) ***Identity sensitive attributes in the patient data:***
      Next, out of all 25 attributes, we took three attributions, i.e., age, gender, and obesity, as sensitive attributes in the data set that need to be protected. Among all 25 features, we believe attributes (age, gender, and obesity) could be used to identify an individual patient from all the patients in the data set.
    (c) ***Choosing privacy models:***
      In the current studies that are going on in patient data privacy while permitting its secondary use (for research-related tasks), both Laplacian and Gaussian mechanisms are being utilized to perturb the patient data (obviously, that exact choice varies according to the setup). Other techniques, e.g., anonymization, data masking, K anonymization, etc., are inappropriate when protecting patients’ data [48]. According to Li et al. [48], k-anonymity is syntactic and weak. At the same time, on the other side, differential privacy is entirely based on algorithms and provides semantic privacy guarantees for the privacy and utility of the data. Similarly, Sweeney demonstrated in [50] that a person’s gender, date of birth, and zip code are enough to identify most Americans.
      She proved this by matching supposedly anonymous healthcare data to public voter records, which allowed her to identify the health records of the Governor of Massachusetts. These types of attacks, known as linkage attacks, stress the need for a robust privacy definition that can withstand attacks using additional knowledge, including knowledge that a data curator may not anticipate. So, in our paper, we came up with an approach where we used both Laplacian and Gaussian mechanisms to assess the suitability of the noise addition method to patient data when using DCMS. Obviously, in DCMSs, there is a great need to tackle privacy and utility of patient data when sharing to DC and DR, so we performed tests having Laplacian and Gaussian mechanisms.
    (d) ***Implement the noise addition method:***
      After choosing both privacy models for patient data perturbation in DCMS, we implement the noise addition method in Python. The code for these experiments is available here *Differential-Privacy-for-DCMS*: https://github.com/Khalid-Muhammad-Irfan/Differntial-Privacy-for-DCMS (accessed on 3 July 2023). We wrote a code snippet using that “patient data CSV file”. Exploiting this implementation, we added random noise to the sensitive attributes (age, gender, and obesity) in the patient data and noted the results at different values of privacy parameter ϵ. The added noise, privacy, and utility effects for the patient data with the different values of ϵ can be seen in Figure 4 and Figure 5.
    (e) ***Evaluating the privacy guarantee:*** After adding noise to the patient-sensitive attributes, i.e., age, gender, and obesity, we evaluated the privacy guarantee of each of the noise addition mechanisms (Laplacian and Gaussian). This step gave us valuable insights into the suitability of methods that can confirm the privacy of the patient data, along with measuring the amount of privacy loss that occurs when the data requester (DR) analyzes the data.
    (f) ***Evaluating the utility of the data:***
      In addition to evaluating the privacy guarantee, we also evaluated the utility of the patient data for the DR. This step involved measuring the benefit of patient data for a researcher who is the ultimate user of patient data.
    (g) ***Comparing employed DP noise addition mechanisms:***
      Finally, in this step, we compared both Laplacian and Gaussian methods for different ϵ values to predict which one provides the best trade-off between privacy and utility of the sensitive patient data.
      So, in a DCMS, the patient’s data (DS) can be protected by adding moderate noise using the Gaussian mechanism before it is shared with the hospital (DC). It is assumed that the DC is collecting data from multiple patients. The DC then accumulates and stores the data in the DCMS database server. When a researcher (DR) requests access to the patient’s data, the DC shares the aggregated data with the researcher. The Y-axis represents the frequency of occurrence of each data point for the attributes (age, gender, and obesity) present in the CSV file. The X-axis displays the attributes (age, gender, and obesity). Dyda and Canonne et al. state in their papers [47,52] that in situations where data of many individuals need to be shared with various entities, the sensitivity of the data is generally significant. However, in cases where only a small number of individuals’ data needs to be shared, the Laplace mechanism is a better choice as it provides stronger privacy guarantees. However, the Gaussian mechanism is more appropriate in scenarios where the data has both high sensitivity and many individuals, such as patient data. In the view of Dong et al. [53], the Gaussian mechanism can provide strong privacy guarantees while adding less noise to the data and maintaining higher utility for the data users. The Gaussian mechanism adds random noise to the data to keep privacy while maintaining the overall utility of the data.
    (h) ***Calculating Root Mean Square Error (RMSE) for both Laplacian and Gaussian Mechanism at different values of*ϵ:**
      Root mean square error (RMSE) is a commonly used metric to evaluate the accuracy of a privacy model in terms of the error it introduces to the data. In this case, we are using RMSE to assess the accuracy of the Laplace and Gaussian mechanisms in adding noise to the sensitive attributes of the patient data. The RMSE measures the difference between the values predicted by the privacy mechanisms and the original values of the sensitive attributes. By plotting the RMSE against the different epsilon values, we evaluated the accuracy of the noise addition mechanisms across a range of privacy levels. We are using RMSE specifically because it provides a way to measure the error in the same units as the data, which makes it easy to interpret the accuracy of the mechanisms.
      Additionally, it is sensitive to positive and negative errors, making it a valuable measure for assessing the precision of models that can under or over-predict values. We calculated the RMSE values against various values of ϵ. This analysis can be seen in Figure 6. Our study evaluated the Laplace and Gaussian mechanisms for obscuring sensitive patient attributes such as age, gender, and obesity. We varied the epsilon values from 0.1 to 20 to determine the optimal level of privacy preservation with reasonable accuracy. By analyzing the RMSE values, we identified the range of epsilon values where the Laplacian and Gaussian methods only moderately perturbed the data attributes. Our findings suggest that the Gaussian mechanism performed better than the Laplace mechanism, resulting in significantly lower RMSE values for the polluted patient data attributes.
  - **Our Findings regarding DP Implications in DCMS:**
    − When the value of ϵ is increased, the amount of noise added by Laplacian and Gaussian mechanisms is reduced. This decrease in noise comes at the cost of decreased privacy protection provided by these mechanisms. However, noise reduction benefits the DR in data analysis. For example, if ϵ=5, less noise is added compared to smaller values of ϵ=1, resulting in more minor discrepancies between the noisy and original data.
    − When random noise is introduced to three attributes (age, gender, obesity) of patient data, both Laplacian and Gaussian mechanisms were used with the same value. Our findings indicate that the Gaussian mechanism is better than the Laplacian, as it adds less noise, making the data more usable for researchers and maintaining the confidentiality of patient data attributes. For instance, when the same ϵ=0.5 value is used for both mechanisms, the Laplacian mechanism adds excessive noise to the patient data attributes, such as age, gender, and obesity, while making data unsuitable for secondary tasks.
    − As the value of ϵ is increased, there is a decrease in RMSE error. This states that the noise added to the data attributes is slightly reduced, resulting in a minor deviation between the original data and the perturbed data and lower privacy.
    − Our findings suggest that for the Gaussian mechanism, adding moderate noise preserves patient data privacy and enables a researcher to analyze this data. A Gaussian mechanism with lower values of ϵ also improves the RMSE values.
    Hence, choosing a concrete epsilon value is a complex task. It requires more careful considerations, depending on the data one wants to pollute using the DP framework.
    In this paper, we demonstrated the possible contribution of differential privacy (DP) after doing simulations with patient data. Later, we will be giving a follow-up work with the above-described scenario where we will study the more specific aspects of patient data perturbations, and ultimately, we will give our findings in the form of a design for the privacy and, at the same time, utility of the patient data in a dynamic consent management system (DCMS).
  - ***Zero Knowledge Proofs (ZKP’s):***
    Goldwasser and colleagues first conceived zero-knowledge proofs [54]; their question was, “How much knowledge should be conveyed to show proof of some knowledge”. With the help of zero-knowledge proofs in DCMS, the data subject could provide aggregated information to the controller without exposing the underlying details. At the same time, the data controller can ensure that the aggregated data is correct (i.e., it corresponds to the aggregation of accurate data generated by certified data generators).
    ZKP could be one of the essential tools that can extend the resilience of DCMS when there is a need for extensive confidentiality while hiding the identities of actors in a DCMS. For instance, the data subject can prove to a data controller that she is a legitimate party to access and share data and consent inside DCMS. In this case, the DS can provide some aggregated information to the DC, and the DC comprehends the validity of the DS; likewise, when a DR has to prove to the DC that he has the right to access data and consent of the DS. DR will be furnishing some aggregated information to DC, and with this information, DC will learn about the legitimacy of DR. More formally, the requirements for ZKP for a DCMS can be stated as follows:
    (a) **Data subject proving the possession of credentials that guarantee that the subject is the genuine entity:**
      Regarding DCMS, zero-knowledge proofs empower the data controller to verify that a data subject knows the discrete logarithms $y_{1}$ and $y_{2}$ of the public values $Y_{1}=y_{1}B$ and $Y_{2}=y_{2}B$ and that they comply with a linear equation
      (2) $$A_{1}{\dot{y}}_{1}+A_{2}{\dot{y}}_{2}=A,$$
      where *A*, $A_{1}$, and $A_{2}$ are public points on G. This is done by not exposing any data concerning $y_{1}$ or $y_{2}$.
      ***Completeness:*** Whenever the aggregated information supplied by the data subject to the data controller stands TRUE, the subject convinces the controller in a DCMS.
      ***Soundness:*** Whenever the aggregated information delivered by the data subject to the data controller exists FALSE, a dishonest data subject will not be capable of persuading the data controller in a DCMS.
      ***Zero-knowledge:*** The data controller will not learn anything from the aggregated information given by the subject, whereas the statement provided by the subject is either true or false.
    (b) **Data requester proving the possession of credentials to get subject’s consented data.**
      Regarding DCMS, zero-knowledge proofs empower the data controller to verify that a data subject knows the discrete logarithms $y_{1}$ and $y_{2}$ of the public values $Y_{1}B$ = $y_{1}B$ and $Y_{2}$ = $y_{2}B$ and that they comply with a linear equation
      (3) $$A_{1}{\dot{y}}_{1}+A_{2}{\dot{y}}_{2}=A,$$
      ***Completeness:*** Whenever the aggregated information delivered by the data requester to the data controller stands TRUE, the data requester convinces the data controller to have the subject’s data in a DCMS.
      ***Soundness:*** Whenever the aggregated information provided by the data requester to the data controller is FALSE, a dishonest data requester will not be able to convince the data controller to have the subject’s data in a DCMS.
      ***Zero-knowledge:*** The data controller will not learn anything from the aggregated information the data requester provides. Still, the statement provided by the data request is either true or false.
      **Contribution applying ZKP to concrete settings in DCMS:***Using Zk-SNARKs in a blockchain-based DCMS:* In addition to the implications mentioned above regarding the use of zero-knowledge proofs in DCMS, we can also see substantial contributions of ZKPs when we apply DCMS to real-life systems, such as patient data/consent sharing with researchers who require patient data for their research assignments.
      **DCMS scenario:** In this context, a patient can be considered as a DS whose data/consent is being shared from a DC with DR. The healthcare organization acts as a centralized entity responsible for receiving, storing, and sharing the DS’s data/consent.
      − ***Decentralizing the DC:***
        Firstly, to avoid a single point of failure in this scenario, decentralization of the DC can be achieved through the hyper ledger fabric blockchain. Since we have known entities in patient data management through DCMS, a modular architecture provided by hyper-ledger fabric would be beneficial. A modular approach can be employed by putting only the DC on the blockchain, with peers on the blockchain network representing the DC, such as endorsing peers and committing peers [55]. In addition, it is essential to note that DC is a healthcare organization that only exists as a virtual entity implemented through a group of hospitals on the fabric blockchain. In a healthcare system built on the hyper ledger blockchain, a group of endorsing peers can manage patient consent and data sharing. The endorsing peers receive patient data/consent, and when a patient provides data/consent, an endorsing peer initiates a transaction, which is then broadcast to the blockchain network. The transaction is then validated by other available endorsing peers using a consensus algorithm, such as PBFT, commonly used in hyper ledger blockchain. This consensus algorithm ensures that most of the network’s peers agree on the transaction’s validity. Once the transaction is validated, it is sent to the committing peers, which update the blockchain with the new transaction.
      − ***Zk-SNARKs in blockchain powered DCMS:***
        Zk-SNARKs (Zero-Knowledge Succinct Non-Interactive Argument of Knowledge) is a cryptographic technique used in blockchain to provide privacy and confidentiality in transactions [56]. Utilizing zk-SNARKs in a blockchain-powered DCMS can guarantee the confidentiality of sensitive patient (DSs) sensitive data while permitting researchers (DRs) to access the necessary patient data for their research tasks. According to Anusuya et al. [56], zk-SNARKs enable the creation of a proof of knowledge that verifies the validity of transmitted data without exposing the actual data. This technology can securely transmit sensitive patient data over a blockchain network while remaining private. In a blockchain-based DCMS, patient data can be encrypted and stored on the blockchain, and zk-SNARKs can be utilized to prove the data’s validity without revealing its contents. This ensures that patient privacy is maintained while enabling the DR to use the data for research. The blockchain can serve as a decentralized and distributed database, with all network participants having access to a copy of the data. Meanwhile, the current studies on zk-SNARKs have outlined the improved efficiency and robust privacy protection when using them with blockchain technology [57].
        Huang et al. [57] offered a method known as ZKchain, which uses zk-SNARKs in blockchain technology to improve privacy and efficiency. Additionally, zk-SNARKs have been successfully applied in developing Zcash, a cryptocurrency known for its robust privacy protections. [58]. All those studies cited in [57,58,59] did not focus on a DCMS scenario, unlike our proposal, which leverages zk-SNARKs in a blockchain-based DCMS. In our approach, zk-SNARKs play a crucial role in ensuring that patient data is securely stored on the blockchain without any reliance on the trustworthiness of the involved parties, i.e., DCs. Zk-SNARKs enable data sharing while keeping patient information confidential. Additionally, zk-SNARKs can verify patient data’s integrity, increasing trust in the research results. This approach can also instill confidence in patients that their data is safe and has not been compromised. Therefore, implementing zk-SNARKs in a blockchain-based DCMS can enhance patient privacy, ensure data security, and facilitate valuable research.
        In this paper regarding data protection in DCMS, we discuss a theoretical approach to the design of a privacy-preserving DCMS. In the future, we plan to study practical implementations that utilize zk-SNARKs within a hyper-ledger fabric blockchain system. This approach would help to decentralize the abstract entity responsible for data control and processing while ensuring the confidentiality of the subject’s data and the integrity of the overall process.
  - **Unforgeability:**
    The dynamic consent management system should be able to provide correct data to honest players, i.e., DS, DC, DA, and DR, even in the presence of adversaries who can attack and change the output. In addition, in a DCMS, the emulation and copying of valid login credentials from any player (i.e., DS, DC, DA, DR) end should be infeasible. In a DCM system, with unforgeability, we mean that the expected output from the DCMS will not change even in the presence of adversaries. In a DCMS, the emulation and copying valid login details/credentials from any actor’s side should be infeasible. We summarize the security goals for DCMS as follows, and we only describe those highly relevant to DCMS. *Cryptographic Security, End-to-End Encryption, Blockchain-Based Security, and Authentication, and Authorization.*
    − ***Cryptographic Security:*** For a DCMS, where a few entities are communicating with each other, and there is an exchange of the data of a data subject, cryptography can be used to have the critical features for the integrity of the data of all the entities while communicating.
    − ***Homomorphic Encryption (HE):***
      According to [60], homomorphic encryption encompasses several categories of encryption procedures to get various analyses of encrypted data. For the confidentiality of the data, HE emphasized that if the processing of the subject’s data is approvingly required, then the sharing and processing of the subject’s data should be accomplished in an encrypted format so that at any level, no one can interpret that sensitive data of a data subject. The outcomes of any analysis remain encrypted, and only the actors in a DCMS can decrypt their data. Furthermore, homomorphic encryption can also be utilized in a DCMS when a data subject desires to disseminate the processing of their data without disclosing personal details in plain text. In the same way, when a data requester needs to have the subject’s data for his research-related tasks, utilizing the HE, the controller can share the subject’s data without revealing personal details of the subject in plain text.
      The computing circumstances would not be capable of comprehending the data and outcomes, which both stay encrypted. Hence, homomorphic encryption facilitates secured computation on an untrusted computing platform, i.e., dynamic consent management systems.
      *More Formally:* A dynamic consent management system may need an add-on homomorphic cryptosystem. For instance, if we utilize Elliptic Curve El Gamal [61], that empowers the effective management of zero-knowledge proofs for correctness [62]. Elliptic Curve El Gamal depends on how hard it is to calculate a discrete logarithm in a particular zone; now, an elliptical curve is a subset of Zp from p large prime. The encoding of a declaration by which m ∈ Zp is.
      (4) $$E_{\Omega }\left(m\right)=\left(rB,mB+r\Omega \right),$$
      where r is uniformly incidental nonce around Zp, *B* remains a reference position regarding the elliptical curve *G*, as Ω is the public key. The additive’s homomorphic property determines that the
      (5) $$E\Omega \left(\alpha m1+\beta m2\right)=\alpha E\Omega \dot{(}m1)+\beta E\dot{\Omega }\left(m2\right)$$
      for messages m1 and m2 and for any scalars α and β. Decrypting encrypted text(rB,mB + rΩ), the owner of associated private key ω (Ω = ωB) multiplies rB and ω yielding ω (rB) = rΩ and subtracts this point from mB + rΩ. The result mB is then plotted back to m using a hash table. For DCMS, symmetric homomorphic encryption schemes could guarantee the confidentiality of subject, controller, and requester data in the presence of unauthorized parties by allowing end-to-end encryption.
    − ***Multi-Party Computation (MPC):***
      Multi-party computation is the underlying domain of cryptography to produce strategies for players to calculate a function over their inputs while keeping them confidential [63]. A DCMS is a perfect scenario to implement secure multi-party computation as the entities would be interacting with each other. Unlike traditional cryptographic tasks, cryptography in secure multi-party computation guarantees the integrity and confidentiality of communication or storage, and the adversary is outside the participants’ system. Cryptography in a secure multi-party computation model protects the actor’s privacy from each other in a DCMS. In the DCMS, we are assuming that all the actors (subject, controller, and requester) can be harmful to each other. Keeping this fact in mind, the secure multi-party computation must be utilized so that all the actors can jointly compute a function over their inputs to the DCMS while keeping those inputs private; hence, their confidentiality is rigorously protected.
      *More formally:* MPC can be expressed in DCMS as we have three players, i.e., DS, DC, and DR, with respective inputs x,y,z indicating their messages in DCMS. Before sending data to the requester, the data controller wants to find out the legitimate requester among them without revealing much information to each other.
    − ***Blockchain-Based Security:***
      Blockchain can impact meaningful results in a DCMS. For instance, while sharing subject data and consent in a DCMS, blockchain can ensure the integrity, availability, and transparency of the shared data and consent. In a DCMS, the data controller is the sole entity that will hold an enormous amount of data. This controller, intrinsically, is a centralized entity. If, for some reason, the server where data and consent of the subjects are being stored is hacked or damaged, then there is a high probability that the data will be gone. So, to overcome this alarming problem, the data controller can be decentralized in DCMS using blockchain technologies.
      Furthermore, the architecture of the DCMS exhibits that only known entities will interact with a DCMS; hence, using a private blockchain is highly recommended where the miners are known, and all the entities in a DCMS should enjoy all the security features. Apart from that, to record all the requests from the data requester in a distributed ledger, the data requester can also be a node in the blockchain and record all the actions from the requester’s end in a permanent ledger of transactions. In the detailed analysis of using blockchain technology to have robust privacy and security in a DCMS, we have elaborated in detail on the Zero-knowledge proof implications of DCMS.

### 6.1. Tension among the Defined Properties

A practical realization may only fulfill some of these prerequisites depending on the underlying business case and the technical restraints. To be more realistic concerning the defined properties in Section 5, one can only achieve some of these properties, which entirely depends on the use case for which these properties will be utilized. For instance, if we take an example where DCMS is going to be used for patient data sharing for secondary usage, then it is very apparent that one of the overhead security properties, “Unforgeability”, underlies blockchain-based security. If we employ blockchain technology for sharing the consented data with the requester, then the data of the subjects would be stored permanently on the blockchain and cannot be removed by any means. At the same time, one of the GDPR’s legislation states that when a subject wants to remove their data, the data management authority must remove the subject’s data from all the storage and servers where that data was stored and shared. Here comes a great tension. This is one of those strains that can hinder achieving all the properties, i.e., confidentiality, unforgeability, availability, and auditability.

### 6.2. Real Life Use-Case in Relation to the Implications of DCMS

**Figure 7:**
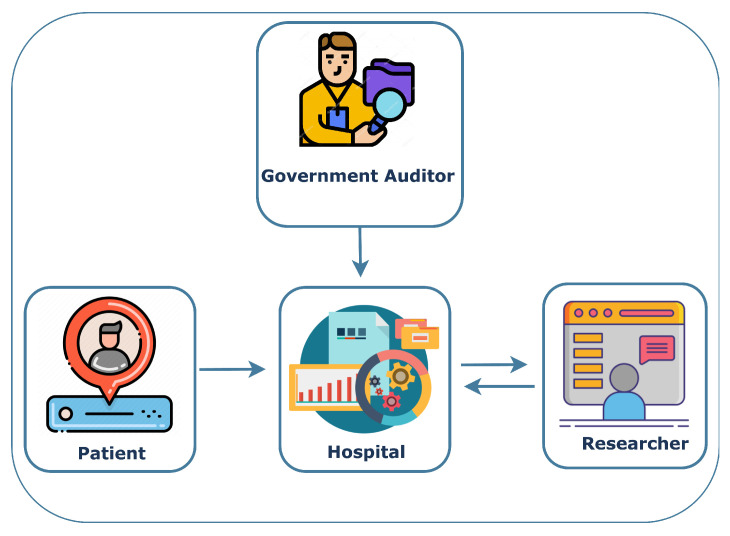
Real-world use-case for the implications of dynamic consent management systems.

In a real-life scenario, following the reforms made by the GDPR [5], along with the implications and the establishment of European Health Data Space in 2022 [64], the data subject has been considered as a patient whose data is being recorded in the hospital upon her checkups. Likewise, the hospital has been regarded as a data controller, a centralized entity that obtains, stores, and processes the patient’s sensitive data after their explicit and unambiguous consent. Furthermore, a data requester/processor has been viewed as a researcher or a group of researchers from a particular university who need patient data for their research-related tasks. Above in Figure 1, we detailed the dynamic consent management system’s entities in general, but here in Figure 7, we are mapping them to patient data/consent management by the hospital and consequently sharing it with the researcher. In Figure 7, Patients refer to the data subjects (DSs) who are sharing their data/consent with the hospital that is acting as a data controller (DC). The government auditor is represented by the data auditor (DA), who will ensure that the hospital (DC) is adhering to the regulations while processing the patient’s (DSs) data/consent. Likewise, the researcher (DR) actually refers to the data requester who acquires patients’ data to perform his research-related assignments.

**Implications of State-of-the-Art Tools and Technologies for DCMS in Healthcare Systems:** We have explored the viability of employing cutting-edge tools and technologies to enhance security and privacy in dynamic consent management systems. To illustrate this, we have presented a real-world scenario where a patient shares her data with a hospital, subject to scrutiny by a data auditor, culminating in data access requests. Through systematic experimentation involving the implications of differential privacy within a dynamic consent management framework, we have concluded that differential privacy holds the potential to bolster privacy for individuals sharing their data in plain-text format. This is especially relevant for cases where patients’ data encompasses sensitive attributes such as age, gender, and obesity—information particularly susceptible to breaches in confidentiality. Utilizing mechanisms such as Laplace and Gaussian within differential privacy contributes to robust privacy protection.

Furthermore, this approach provides researchers, either the end-users or financiers of the data, with satisfactory outcomes when querying for such data. Our discussion on the role of Differential Privacy (DP) which has been demonstrated in detail in Section 6, and in Figure 3, underscores its capacity to yield improved results in the patient data/consent management context, rendering it a superior replacement for the current infrastructure. Similarly, the decentralization of the data controller entity in dynamic consent management, particularly in healthcare setups, can be achieved by redistributing the role to multiple entities represented by hospitals. These entities participate in the sharing and processing of sensitive data of subjects. To ensure heightened privacy, we advocate for implementing privacy-enhancing technologies such as homomorphic encryption, multiparty computation, and similar methodologies. These measures empower subjects to share encrypted data, obviating the need to expose raw data to the controller, data auditor, and data requester. Lastly, judicious deployment within healthcare contexts can yield meaningful outcomes when applying zero-knowledge proofs. By minimizing data sharing among various stakeholders, using zero-knowledge proofs can reinforce the robustness of dynamic consent management systems, mainly when applied to healthcare settings as explained in Section 6. This aligns with the data subject rights delineated in the GDPR.

## 7. Discussion

The main objective of this research is to highlight the severe limitations in the existing state-of-the-art dynamic consent management systems by proposing a sequence of resilient properties to highlight the robustness of these systems. Meanwhile, in the current studies, it has been found that much attention has been given to developing dynamic consent management systems. In contrast, none of the studies highlighted security and privacy issues, particularly data protection issues for the involved entities, i.e., the data subject, controller, auditor, and data requester. By giving a set of properties, we defined them formally in each actor’s interaction context. We gave these definitions a preciseness in making a clear distinction among the informal descriptions of these definitions in the existing literature.

Our contribution was the formal and precise description of these definitions. Apart from that, we have discussed critically how a dynamic consent management system will behave when there are no attacks on it and how our defined properties will safeguard the data of the involved entities when an insider or outsider foe tries to comprise the integrity and confidentiality of these dynamic consent management systems. In the last part of this research, we have trickled down our investigative approach, and under the defined properties like Confidentiality, Unforgeability, Availability, and Auditability, we have proposed the use of advanced tools and techniques that can increase the robustness of these dynamic consent management systems. This study not only highlighted the implacability of state-of-the-art tools and techniques; instead, we practically implemented a couple of strategies to prove the validity of our argument. For this purpose, we have also tested the possibility of using differential privacy mechanisms, i.e., Laplacian and Gaussian with dynamic consent management setting where a data subject is essentially polluting her data/consent by adding random noise.

**Figure 8:**
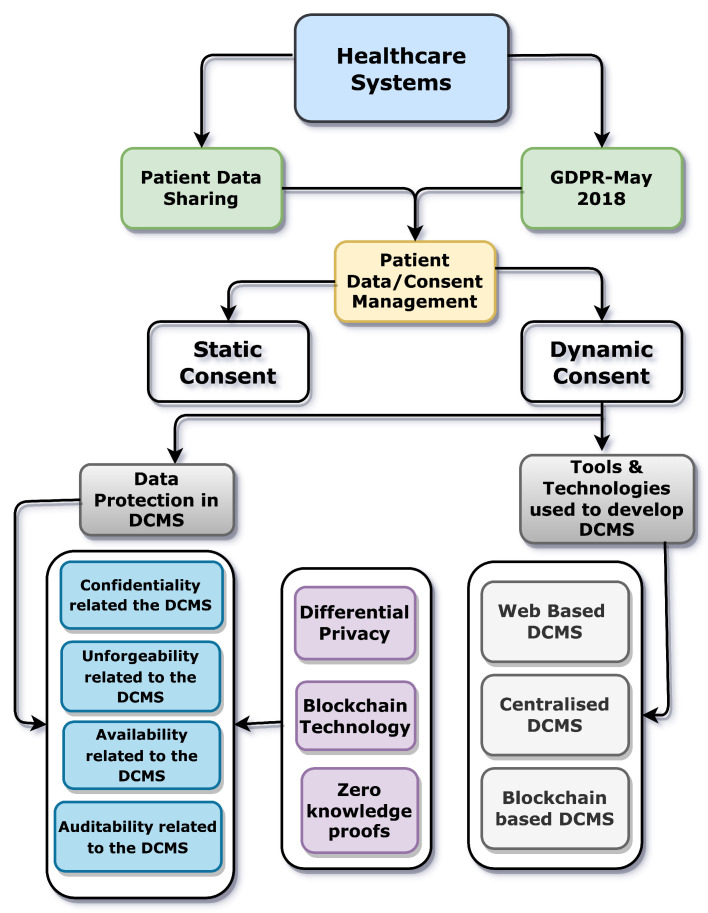
Taxonomy of dynamic consent management systems in relation to the existing research and contributions of this work.

After that, the data controller aggregates noisy data to invade an active link between a data subject and a particular data attribute. Finally, we have discussed how the trade-off between privacy and the utility of the subject’s data/consent could be relaxed. Likewise, using blockchain applications to store and process the subject’s sensitive data/consent, we have discovered the use of blockchain technology only to decentralize the abstract actor, the data controller. For this decentralization, we have suggested using a hyper-ledger fabric blockchain that will only allow the known data controllers to interact with each other. Finally, using the definition of unforgeability, we have found the applications of zero-knowledge proofs, and one of the promising applications of zero-knowledge proofs is ZK-SNARKS’s relation to dynamic consent management systems when blockchain and ZK-SNARKS are used together. The taxonomy of the dynamic consent management systems research concerning the existing state of the art and the contribution made in this work can be seen in Figure 8. Following are the significant open research challenges along with the limitations of this study:

In this research article, we have mainly addressed the security and privacy issues associated with the software systems that, in turn, are used for data and consent collection, storage, and processing. We will highlight the limitations and open challenges that we have found after carrying out this work:

(a) **Decentralization of Data Controller**: In our contributions to this article, we have realized the potential issues that are linked with the abstract entity “Data controller”. Instead of all these four entities, i.e., “DS, DC, DA, and DR”, only the data controller must be decentralized using permissioned/private blockchains such as hyper-ledger fabric. It is clear that when these dynamic consent management systems (DCMSs) are utilized in practical scenarios such as in hospital management systems to tackle the data protection issues for the data subject, there will be plenty of data controllers, and only these controllers will be on the blockchain. We did not explore this challenge in this work, which stands as a limitation of our study, but for future researchers, this is an obvious open problem.
(b) **Zero-knowledge Proofs:** In this work, we have theoretically elaborated on the relevance of zero-knowledge proofs, but we did not perform any experimentation to have some results. We have elaborated on the requirements for applying ZKPs to the dynamic consent management systems. This is also another limitation of our study in the sense that our work provides suggestions to use blockchain, preferably private blockchain, along with the promising application of zero-knowledge proofs, that is, zk-snarks. For future research, this could be another open challenge to test the implacability of one of the promising applications of zero-knowledge proofs to have more precise and accurate results where there should be a minimum amount of data-sharing among all these four actors of dynamic consent management systems.
(c) **Cryptographic Techniques in Relation to DCMSs:** In our work, we studied mainly the implications of privacy-preserving technologies (PETs) such as Homomorphic Encryption and Multiparty Computations that certainly are much more relevant to dynamic consent management systems, especially when two parties need to interact with each other in a privacy-preserving manner. We have suggested the suitability of these cryptographic tools concerning DCMS with some concrete feasibility assessment. This is another open challenge for further researchers and is one of the limitations of our study.

## 8. Conclusions, and Future Work

In this work, we have discussed natural security and privacy properties for dynamic consent management systems that had been somewhat overlooked in prior work. We have drawn up the importance of the dynamic consent management system’s security and privacy aspects in the context of the subject’s data-sharing with a data requester in secondary utilization. We have practically elaborated to achieve one of the essential objectives of developing sustainable e-healthcare services by employing advanced security and privacy tools and techniques such as differential privacy, blockchains, and cryptographic primitives that can ascertain the robustness of the dynamic consent management systems. We leave to future work the design and analysis of dynamic consent management systems that enjoy security and privacy by design. Above all, this study provides useful theoretical and practical background for further research to be carried out in the future. Future directions to investigate are as follows:

(a) We have studied the potential of one of the most promising applications of zero-knowledge proofs, which is “Zero-Knowledge Succinct Non-Interactive Argument of Knowledge” (zk-SNARK). We have found that the data controller is placed onto the hyper-ledger fabric blockchain while zk-SNARK is used by the parties, i.e., use of zk-SNARK by the data subject while sending data/consent to the data controller. Use of zk-SNARK by the data controller allows the data auditor to audit the operations made by the data controller, and finally, use of zk-SNARK by the data requester while asking for the subject’s data and consent. In all these interactions, each party can share proof, and the other party will be convinced that the other party is the legitimate one to have the required data. Future researchers can make use of our study. They may produce more fruitful results that can mature the practical viability of dynamic consent management systems, especially in those real work scenarios where sensitive data must be shared, i.e., sharing patient data with the researcher to gain good research results.
(b) In this work, we have tested the validity of differential privacy for dynamic consent management systems and found its amazing implications with the core of dynamic consent management systems. For future research, it may be fruitful to use both the differential privacy mechanisms to have more results and develop a study on top of this work.

## Figures and Tables

**Table 1:** List of Acronyms.

Acronym	Description
DS	Data Subject
DC	Data Controller
DA	Data Auditor
DR	Data Requester
DCMS	Dynamic Consent Management System
PrivKdcms	Private-key encryption scheme experiment within DCMS
Π	Protocol used within the context of DCMS comprising some algorithms
n	Security parameter
Pr[…]	The probability of the event
PrivKdcmsA	Private-key encryption scheme experiment involving a specific participant within DCMS
A	Probabilistic polynomial-time adversaries, i.e., DC, DA, DR
gen	The key generation algorithm
sign	The message signing algorithm
vrfy	The key verification algorithm
access	Access algorithm in DCPrivKdcms experiment
share	The data sharing algorithm
negl	A negligible function
IND	Security property of DCMS
pk	Public key
sk	Secret key
k	Encryption key used to encrypt the messages
Enc	Encryption function that takes a message
DP	Differential Privacy
ZKPs	Zero Knowledge Proofs
G	A generator point on an elliptic curve
RMSE	Root Mean Square Error
zk-SNARK	Zero-Knowledge Succinct Non-Interactive Argument of Knowledge
HE	Homomorphic Encryption
MPC	Multiparty Computation
E Ω (m)	The encoding of a message or value ’m’ using a public key Ω
r	A randomly chosen nonce
Zp	The set of integers modulo a prime number p
B	A reference point on an elliptic curve G
GDPR	General Data Protection Regulation
EHDS	European Health Data Space
PBFT	Practical Byzantine Fault Tolerance
HIPAA	Health Insurance Portability and Accountability Act
OutputForge A	Output associated with adversary *A*
$1^{n}$	A unary representation 1
c	The resulting cipher-text
m	Plain-text message that is being encrypted

**Table 2:** Analysis of the related works in DCMS (why DCMSs presented in prior results are not good).

Reference	Confidentiality	Unforgeability	Availability	Auditability
[12]	×	×	×	×
[24]	×	×	×	×
[25]	Σ	Σ	Σ	ϕ
[26]	×	×	×	×
[27]	×	×	×	ϕ
[28]	×	×	×	Σ
[29]	×	×	×	ϕ
[30]	×	×	×	ϕ
[31]	×	ϕ	×	ϕ
[13]	×	×	×	ϕ
[32]	×	×	×	ϕ

× Shows that the article did not consider our defined properties concerning the proposed DCMS. ϕ Shows that a paper claims that our described property with the given DCMS is being achieved, but we could not find sufficient evidence in the paper that this is true. Σ Shows that the description of our defined property about the proposed DCMS is vague, and we cannot understand it.

**Table 3:** Motivation behind considering security and privacy properties of DCMSs.

References	Comments by the Authors
[33,35]	Various technological barriers exist to DCMS implementation; security and privacy concerns are significant. This paper elaborates on the security and privacy of the DCMS question as follows: How can security practices be addressed to ensure patient privacy in DCMS? Traceability and transparency: How can patient preferences be tracked across complex landscapes where patient data are shared? Interoperability: How can a DCMS be integrated effectively into clinical genetics environments?
[36]	The utilization of DCMS for administering the right to access scientific genetics data can be similar to data security and privacy problems, like the other software used in this domain. In particular, the confidentiality, integrity, and availability of personal information concerning consent are crucial.
[37,38]	Software sellers/developers must be obliged to respect both optional and needed security and privacy requirements in the product, which would be dynamic consent software.
[39,40]	Different security administrations may put security and privacy concerns in circumstances that could manufacture data from a clinical perspective but store or transfer it to research-related tasks.
[41]	Transferring clinical data to high-performance computing clusters outside the hospital network is commonplace today. In such a situation, the security and privacy concerns are not unique to dynamic consent. Using DCMS, the increased mobility and volumes of data access in a setting stress the importance of addressing security problems purposefully.
[42]	Cross-organization or cloud-based networks of access to personal data can challenge existing security roles. Moreover, this also raises questions about the competence of the interested parties and the authority they belong to. A DCMS must also bear a trusted identity management process, including identity proofing, credentialing, authentication, and authorization, to confirm the security and privacy of patient data.
[43]	The EU health data space committee agrees on the security and privacy enhancement in the current systems that share patient data for research/secondary use. In future designs, patients will be at the system’s heart and have complete control over their data. The Commission proclaimed the utilization and advances in cybersecurity for health data-sharing practices.
[44]	The EU made another initiative to enhance the technical realization of DCMS to enable citizens to give informed consent and manage access rights and permissions to personal health data for sharing unique, identifiable health data with healthcare professionals and others.

## Data Availability

The data used in this research can be obtained from the corresponding authors upon request.
